# Optimal Pulse Design for Dissipative-Stimulated Raman Exact Passage

**DOI:** 10.3390/e25050790

**Published:** 2023-05-12

**Authors:** Kaipeng Liu, Dominique Sugny, Xi Chen, Stéphane Guérin

**Affiliations:** 1 Laboratoire Interdisciplinaire Carnot de Bourgogne, CNRS UMR 6303, Université de Bourgogne, BP 47870, 21078 Dijon, France; 2 International Center of Quantum Artificial Intelligence for Science and Technology (QuArtist), Department of Physics, Shanghai University, Shanghai 200444, China; 3 Department of Physical Chemistry, University of the Basque Country UPV/EHU, Apartado 644, 48080 Bilbao, Spain; 4 EHU Quantum Center, University of the Basque Country UPV/EHU, Barrio Sarriena, s/n, 48940 Leioa, Spain

**Keywords:** quantum control, quantum system driven by an external field

## Abstract

Quantum control of lossy systems is known to be achieved by adiabatic passage via an approximate dark state relatively immune to loss, such as the emblematic example of stimulated Raman adiabatic passage (STIRAP) featuring a lossy excited state. By systematic optimal control study, via the Pontryagin maximum principle, we design alternative more efficient routes that, for a given admissible loss, feature an optimal transfer with respect to the cost defined as (i) the pulse energy (energy minimization) or (ii) the pulse duration (time minimization). The optimal controls feature remarkably simple sequences in the respective cases: (i) operating far from a dark state, of π-pulse type in the limit of low admissible loss, or (ii) close to the dark state with a counterintuitive pulse configuration sandwiched by sharp intuitive sequences, referred to as the intuitive/counterintuitive/intuitive (ICI) sequence. In the case of time optimization, the resulting stimulated Raman exact passage (STIREP) outperforms STIRAP in term of speed, accuracy, and robustness for low admissible loss.

## 1. Introduction

Quantum control often faces losses or noise that have to be circumvented in order to make operational the modern quantum technologies, such as in quantum information processing [1,2]. Two intuitive opposite strategies have been generally developed: (i) operating sufficiently fast [3], i.e., in a timescale in which the loss does not act, or (ii) operating adiabatically along a dark state, which is by construction immune to loss, as in the emblematic example of stimulated Raman adiabatic passage (STIRAP) [4,5,6,7]. The first strategy is an intensive area of research, including optimal control [2,8], shortcut to adiabaticity [3], single-shot shaped pulse [9,10,11], and robust optimization [12,13,14,15]. Optimal control theory (OCT) is a powerful tool to mitigate intensities of the control pulses and speed up the evolution allowing one in principle to reach the ultimate bounds in the system (often referred to as the quantum speed limit when optimal time is considered) [16]. Besides numerical implementation of OCT, such as gradient ascent algorithms (GRAPE) [17], the Pontryagin maximum principle (PMP) [8,18,19,20] allows analytic or semi-analytic derivation of the optimal controls (typically with respect to time or energy) in transforming the initial infinite-dimension control problem into a finite-dimension problem. In the second strategy, the process is, however, approximative due to adiabatic principles, but it is also affected by losses, since the dark state is only an approximation of the dynamics for a realistic finite time, i.e., not strictly adiabatic, process.

In this paper, we determine optimal routes in a Λ-system, where the upper excited state, coupled to both ground states, is lossy, yielding necessarily to a lossy dynamics when the two ground states are not directly coupled. We follow an inverse-engineering strategy: we start by assuming a total loss (at the end of the dynamics) that is defined a priori as an admissible loss. We then determine using the PMP the optimal dynamics with respect to a given cost (the pulse duration or the energy) that will reach the target state and realize the admissible loss. As a consequence, the transfer to the target state is incomplete due to the loss, but the fidelity of the transfer is a priori known and controlled. We then refer this process to an optimal dissipative stimulated Raman exact passage (optimal dissipative STIREP), similarly to its definition in the non-linear case [21].

When the pulse energy is considered as cost (for a given pulse duration), the optimal dynamics operates with a relatively strong field far from a dark state. In the limit of low admissible loss, one can reformulate the problem as the control of a planar pendulum and an analytic expression of the coinciding pulses, as a sine Jacobi elliptic function, is provided.

On the other hand, when the pulse duration (with constrained pulse amplitudes) is taken as cost to minimize, we explicitely determine the optimal dynamics. A key result of this work is established in the limit of low admissible loss: we derive a faster, more accurate and more robust dynamics than the traditional STIRAP. The resulting optimal pulse sequence is remarkably simple, featuring a slow counterintuitive order, reminiscent of the STIRAP sequence, but sandwiched by two sharp intuitive sequences.

The paper is organized as follows: in Section 2, we define the system. In Section 3 and Section 4, we determine the energy-optimal and time-optimal dissipative STIREP processes, respectively. We conclude in Section 5, where a comparative analysis and in particular the robustness of the derived optimal processes are provided. Appendices with the details of the calculations complete the paper.

## 2. Definition of the Lossy-Driven Raman System

We consider a three-level Λ-system, in which the Hamiltonian in rotating wave approximation (in units such that ℏ=1) is given in the basis of the states {|1〉,|2〉,|3〉} by:

(1) $$H_{\Gamma }\left(t\right)=\left[\begin{array}{ccc} 0 & u_{p}\left(t\right) & 0\\ u_{p}\left(t\right) & -i\Gamma /2 & u_{s}\left(t\right)\\ 0 & u_{s}\left(t\right) & 0 \end{array}\right],$$

where $u_{p}$ and $u_{s}$ are the pump and Stokes controls, respectively (corresponding to half of the traditional Rabi frequencies), with the lossy upper state |2〉, via the dissipation rate Γ. Instead of analyzing such a complicated lossy system requiring specific adaptation of the cost [22], large dissipation [23], or assumption on the controls [24], we consider an alternative procedure to treat approximately but accurately the problem in the situation of interest having a relatively low dissipation rate without adapting the cost, nor restricting the controls. From the unlossy system $H\equiv H_{\Gamma =0}$, the effects of the loss are taken into account at the second order perturbation theory from the knowledge of the state amplitude of state |2〉 (in absence of dissipation):

(2) $$P_{\mathrm{loss}}:=1-\left(\right|c_{\Gamma ,1}{|}^{2}+|c_{\Gamma ,2}{|}^{2}+|c_{\Gamma ,3}{|}^{2})\approx \Gamma \int_{t_{i}}^{t_{f}}dt{\left|c_{2}\right|}^{2},$$

where $t_{i}$ ($t_{f}$) is the initial (final) time, the state amplitude of state |j〉 is denoted $c_{\Gamma ,j}$ in presence of the dissipation, and $c_{j}$ when Γ=0, respectively. This simplification can be shown to be numerically accurate for a low enough ratio $\Gamma /u_{\max}≲0.1$, where $u_{\max}$ is the peak value of the pulses. We consider that the pulses are exactly resonant because they produce the most efficient two-photon coupling [25].

We denote the state solution $|\psi \rangle ={\left[c_{1},c_{2},c_{3}\right]}^{T}$. We use the alternative notation $x_{1}\equiv c_{1}$, $x_{2}\equiv ic_{2}$, and $x_{3}\equiv -c_{3}$ so that all the elements of the solution $|x\rangle \equiv {\left[x_{1}\left(t\right),x_{2}\left(t\right),x_{3}\left(t\right)\right]}^{T}$ are real when we consider the initial (real) state $c_{1}\left(t_{i}\right)=1$, $c_{2}\left(t_{i}\right)=c_{3}\left(t_{i}\right)=0$, and the time-dependent Schrödinger Equation (TDSE) becomes:

(3) $$\frac{d}{dt}|x\rangle =\left[\begin{array}{ccc} 0 & -u_{p}\left(t\right) & 0\\ u_{p}\left(t\right) & 0 & -u_{s}\left(t\right)\\ 0 & u_{s}\left(t\right) & 0 \end{array}\right]|x\rangle$$

satisfying $x_{1}^{2}+x_{2}^{2}+x_{3}^{2}=1$.

Throughout the paper, we consider an admissible loss, which is, according to (2), characterized by:

(4) $$\int_{t_{i}}^{t_{f}}dt\;x_{2}{\left(t\right)}^{2}=A\approx P_{\mathrm{loss}}/\Gamma ,$$

where *A*, the time area of the population in state |2〉, is a given constant, referred to as the (total) normalized loss (with respect to Γ). The dynamics is determined with the non-lossy Hamiltonian $H_{\Gamma =0}$ and the expected loss is, thus, given by $P_{\mathrm{loss}}\approx A\Gamma$ if one assumes $\Gamma /u_{\max}≲0.1$.

## 3. Energy-Optimal Dissipative STIREP

### 3.1. Construction of the Pseudo-Hamiltonian and Derivation of the Equations of Motion from PMP

The goal of the control is to steer the system from $x_{1}\left(t_{i}\right)=1$ to $x_{3}\left(t_{f}\right)=1$ (chosen as the target; similarly, we could have chosen $x_{3}\left(t_{f}\right)=-1$) in a fixed time $T=t_{f}-t_{i}$ while minimizing the energy of the controls:

(5) $$J=E\equiv \int_{t_{i}}^{t_{f}}\left(u_{p}^{2}+u_{s}^{2}\right)dt,$$

under the constraint (4).

To take into account constraint (4), we augment the dimension of the system with a new coordinate y(t) such that:

(6) $$\dot{y}=x_{2}{\left(t\right)}^{2},$$

of initial $y(t_{i})=0$ and final value:

(7) $$y\left(t_{f}\right)=\int_{t_{i}}^{t_{f}}dt\;x_{2}{\left(t\right)}^{2}\equiv A.$$

The constraint (4) reduces, thus, to a boundary problem on *y*.

Similarly to the unconstraint optimization case [8], it is convenient to use angle coordinates, which simplify the representation of the dynamics from three components to two angles:

(8) $$\begin{array}{c} x_{1}=\cos\varphi \cos\theta ,\;x_{2}=\sin\varphi ,\;x_{3}=\cos\varphi \sin\theta \end{array}$$

with the initial conditions $\varphi (t_{i})=0$, $\theta (t_{i})=0$. The equations of the dynamics (3), complemented by (6), can be simplified as:

(9) $$\begin{array}{cc} \dot{\varphi } & =v_{p}\equiv f_{1}\left(v_{p}\right), \end{array}$$

(10) $$\begin{array}{cc} \dot{\theta } & =-v_{s}\tan\varphi \equiv f_{2}\left(\varphi ;v_{s}\right), \end{array}$$

(11) $$\begin{array}{cc} \dot{y} & =\sin^{2}\varphi \equiv f_{3}\left(\varphi \right), \end{array}$$

after a rotation on the control fields:

(12) $$\left[\begin{array}{c} v_{p}\\ v_{s} \end{array}\right]=\left[\begin{array}{cc} \cos\theta & -\sin\theta \\ -\sin\theta & -\cos\theta \end{array}\right]\left[\begin{array}{c} u_{p}\\ u_{s} \end{array}\right],$$

leading to an invariant cost on the new field variables since $u_{p}^{2}+u_{s}^{2}=v_{p}^{2}+v_{s}^{2}$. According to the PMP [18,26,27], the minimization of the energy (5):

(13) $$E=\int_{t_{i}}^{t_{f}}\left[u_{p}^{2}\left(t\right)+u_{s}^{2}\left(t\right)\right]\;dt=\int_{t_{i}}^{t_{f}}\left[v_{p}^{2}\left(t\right)+v_{s}^{2}\left(t\right)\right]\;dt$$

leads to the control Pontryagin Hamiltonian (see Appendix A, where we have considered the standard choice $p_{0}=1/2$):

(14) $$H_{c}=\lambda_{\varphi }v_{p}-\lambda_{\theta }v_{s}\tan\varphi +\mu \sin^{2}\varphi -\frac{1}{2}\left(v_{p}^{2}+v_{s}^{2}\right)$$

where $\Lambda ={\left[\lambda_{\varphi },\lambda_{\theta },\mu \right]}^{T}$ is the co-state gathering the conjugate momenta of φ, θ, and *y*, respectively. The Hamilton equations lead to the equations of motion (9) and (10) and to:

(15) $${\dot{\lambda }}_{\varphi }=\frac{v_{s}}{\cos^{2}\varphi }\lambda_{\theta }-\mu \sin\left(2\varphi \right),\;{\dot{\lambda }}_{\theta }=0,\;\dot{\mu }=0.$$

This implies that $\lambda_{\theta }$ and μ are constants of motion. The maximization condition of the PMP gives:

(16) $$\begin{array}{c} \frac{\partial H_{c}}{\partial v_{p}}=\lambda_{\varphi }-v_{p}=0,\;\frac{\partial H_{c}}{\partial v_{s}}=-\lambda_{\theta }\tan\varphi -v_{s}=0, \end{array}$$

which yields:

(17) $$v_{p}=\lambda_{\varphi },\;v_{s}=-\lambda_{\theta }\tan\varphi ,$$

leading to:

(18) $$H_{c}=\frac{1}{2}\left(\lambda_{\varphi }^{2}+\lambda_{\theta }^{2}\tan^{2}\varphi \right)+\mu \sin^{2}\varphi =\frac{1}{2}\left(v_{p}^{2}+v_{s}^{2}\right)+\mu \sin^{2}\varphi ,$$

which features an effective autonomous system (i.e., explicitly time-independent, since $H_{c}$ depends only on the dynamical variables and their conjugate momenta). The equations of motion finally read:

(19) $$\begin{array}{cc} \; & \dot{\varphi }=\lambda_{\varphi }, \end{array}$$

(20) $$\begin{array}{cc} \; & \dot{\theta }=\lambda_{\theta }\tan^{2}\varphi , \end{array}$$

(21) $$\begin{array}{cc} \; & \dot{y}=\sin^{2}\varphi , \end{array}$$

(22) $$\begin{array}{cc} \; & {\dot{\lambda }}_{\varphi }=-\lambda_{\theta }^{2}\frac{\sin\varphi }{\cos^{3}\varphi }-\mu \sin\left(2\varphi \right), \end{array}$$

(23) $$\begin{array}{cc} \; & {\dot{\lambda }}_{\theta }=\dot{\mu }=0 \end{array}$$

with the boundary conditions (for a complete population transfer from state |1〉 to state |3〉):

(24) $$\begin{array}{cc} \; & \varphi \left(t_{i}\right)=0,\;\theta \left(t_{i}\right)=0,\;y\left(t_{i}\right)=0, \end{array}$$

(25) $$\begin{array}{cc} \; & \varphi \left(t_{f}\right)=0,\;\theta \left(t_{f}\right)=\pi /2,\;y\left(t_{f}\right)=A. \end{array}$$

### 3.2. Construction of the Optimal Trajectories

We assume a monotonically increasing θ, implying $\lambda_{\theta }>0$. Since $H_{c}$ features an effective autonomous system, we can determine the optimal trajectory by quadrature using $H_{c}=h=const.$:

(26) $$\begin{array}{cc} \dot{\varphi } & =\lambda_{\varphi }=\pm \sqrt{2h-\sin^{2}\varphi \left(2\mu +\frac{\lambda_{\theta }^{2}}{\cos^{2}\varphi }\right)}, \end{array}$$

(27) $$\begin{array}{cc} \frac{d\varphi }{d\theta } & =\frac{\dot{\varphi }}{\dot{\theta }}=\pm \frac{\sqrt{2h-\sin^{2}\varphi \left(2\mu +\frac{\lambda_{\theta }^{2}}{\cos^{2}\varphi }\right)}}{\lambda_{\theta }\tan^{2}\varphi }. \end{array}$$

We can assume φ≥0 (which is satisfied for $u_{p}$ and $u_{s}$, both positive), $t_{i}\equiv 0$, $t_{f}\equiv T$, and $\dot{\varphi }\left(t=T/2\right)=0$ by symmetry, leading to $\varphi \left(T/2\right)\equiv \varphi_{0}$ maximum at t=T/2, and positive (negative) branch of (26) and (27) for t∈[0,T/2], φ increasing from 0 to $\varphi_{0}$ (t∈[T/2,T], φ decreasing from $\varphi_{0}$ to 0).

As detailed in Appendix A, we first integrate Equation (26) and obtain sinφ (A24) as a function of an elliptic sine of time 0≤t≤T/2:

(28) $$\begin{array}{c} \sin\varphi =\sin\varphi_{0}\;\mathrm{sn}\left(2K\left(m\right)\;t/T,m\right), \end{array}$$

where the set of the three parameters $\left\{\mu ,\lambda_{\theta },h\right\}$ is replaced by the set $\left\{m,\varphi_{0},T\right\}$ with the following correspondence:

(29) $$h=\frac{2K^{2}\left(m\right)}{T^{2}}\sin^{2}\varphi_{0},\;\mu =\frac{mh}{\sin^{4}\varphi_{0}},\;\frac{\lambda_{\theta }^{2}}{2h}=\frac{\sin^{2}\varphi_{0}-m}{\sin^{2}\varphi_{0}\tan^{2}\varphi_{0}}.$$

These are determined in order to satisfy the boundary conditions for a given normalized loss *A*.

In order to take into account the boundary condition $y(t_{f})=A$ of (21), sinφ has to satisfy:

(30) $$\int_{0}^{T/2}dt\;\sin^{2}\varphi \left(t\right)=\frac{A}{2}.$$

From the integral of the Jacobi elliptic function in (28), we obtain:

(31) $$\frac{A}{2}=\sin^{2}\varphi_{0}\;\frac{T}{2K(m)}\;\frac{K(m)-E(m)}{m},$$

where E(m) is the complete elliptic integral of the second kind. This gives for the optimal unconstrained system m=0 [8]:

(32) $$A\left(m=0,\varphi_{0}=\pi /3\right)=\frac{3T}{8}.$$

We next integrate Equation (27), as detailed in Appendix B. Imposing by symmetry that $\theta (\varphi_{0})=\pi /4$ leads to the condition:

(33) $$\begin{array}{cc} \; & \frac{\pi }{4}=+\frac{\sqrt{\sin^{2}\varphi_{0}-m}}{\sqrt{1-m}\;\tan\varphi_{0}}\left[-K\left(\frac{m}{m-1}\right)+\frac{\Pi \left(-\tan^{2}\varphi_{0}\frac{m}{m-1}\right)}{\cos^{2}\varphi_{0}}\right], \end{array}$$

which gives an implicit relation between $\sin\varphi_{0}$ and *m*.

For a given value of loss *A* [below 3/8 (32), the latter corresponding to the optimal unconstrained system], we aim at finding the optimal couple of parameters *m* and $\varphi_{0}$, simultaneously satisfying Equations (31) and (33). The obtained data are shown in Table 1.

One can conclude that for a decreasing admissible loss *A*, *m* decreases and $\varphi_{0}$ decreases to π/4, both monotonically since the right-hand side goes to $\varphi_{0}$ when m→−∞. The minimum peak value of φ(t) is, thus, π/4, asymptotically reached in the limit of no admissible loss, A→0.

### 3.3. Derivation of the Pulses and the Dynamics

The original controls $u_{p}$ and $v_{p}$ are obtained by reversing Equation (12), where θ is given by Equations (A31) and (A32), and $v_{p}$, $v_{s}$ by Equation (17) with φ obtained from Equation (28) and the correspondence (29).

**Figure 1:**
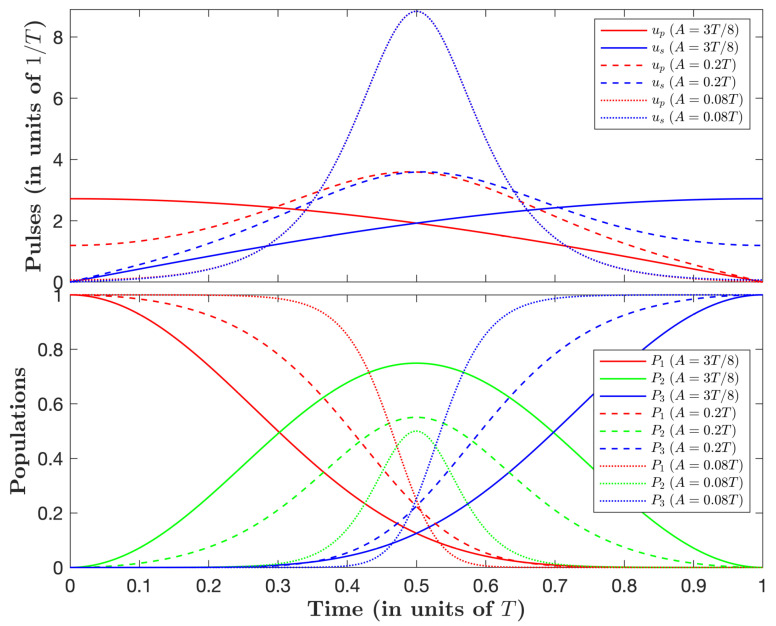
Shapes of the controls $u_{p}\left(t\right)$ and $u_{s}\left(t\right)$ and the corresponding (non-lossy) population dynamics, $P_{j}=x_{j}^{2}$, for A=3T/8 (solid lines), A=0.2T (dashed lines), and A=0.08T (dotted line), with *T* being the duration of the process. The pulses appear as fully overlapping at the scale of the figure in the case A=0.08T.

From the values *m* and $\varphi_{0}$ shown in Table 1, one can derive the controls and determine numerically the corresponding population dynamics. Figure 1 shows such dynamics for three typical values of *A*.

We observe that the pump and Stokes controls operate in the so-called intuitive order (first pump and next Stokes), and that they get closer and coincide more and more with a larger peak for decreasing *A*. One can notice in Figure 1 that the pump and Stokes pulses are almost already undistinguishable at the scale of the figure already for A=0.08T.

We determine that, in the limit of a small normalized loss *A*, i.e., A≲0.08T, corresponding to a large negative *m* and $\varphi_{0}=\pi /4$, the pulses appear as fully overlapping of sine Jacobi elliptic function form:

(34) $$u_{p}\left(t\right)=u_{s}\left(t\right)=\frac{\sqrt{-2m}K\left(m\right)}{T}\;\mathrm{sn}\left(\frac{2K(m)t}{T},m\right)$$

with *m* solution of Equation (31):

(35) $$A=\frac{T}{2}\frac{K(m)-E(m)}{mK(m)},$$

which is well approximated by:

(36) $$m\approx -\frac{1}{2^{4}}e^{T/A}.$$

In this limit, the peak of the pulses is well approximated by:

(37) $$\begin{array}{cc} \max\left(u_{p}\left(t\right)\right)=\max\left(u_{s}\left(t\right)\right) & =\frac{\sqrt{-2m}K\left(m\right)}{T}\approx \frac{1}{\sqrt{2}A}, \end{array}$$

the area of each pulse is:

(38) $$\int_{0}^{T}u_{p}\left(t\right)dt=\int_{0}^{T}u_{s}\left(t\right)dt=\frac{\pi }{\sqrt{2}},$$

and the generalized Rabi frequency pulse area:

(39) $$2\int_{0}^{T}\sqrt{u_{p}^{2}\left(t\right)+u_{s}^{2}\left(t\right)}\;dt=2\pi ,$$

which has to be compared to the optimal Rabi frequency pulse area, which is $\sqrt{3}\pi$ (the counterpart of the π-pulse for Λ systems), obtained in absence of constraint on loss [8].

We conclude that the present energy-optimal pulse operates far from a dark state, of π-pulse type, in the sense that it is relatively close to the optimal Rabi frequency pulse area.

### 3.4. Comparison with Standard STIRAP and Parallel STIRAP

The energy of the derived optimal STIREP pulses with a low normalized loss A≃0.036T (featuring almost overlapping pulses) is compared to that of the standard STIRAP with Gaussian pulses in a situation giving the same normalized loss *A*. We obtain the energy $E≃90\frac{\hbar }{T}$ for STIRAP, almost twice larger than energy-optimal STIREP $E≃56\frac{\hbar }{T}$. The optimal pulses feature a shorter duration, compared to the long adiabatic process of STIRAP, and are more intense with the peak $u_{\max}≃19.6/T$ three times larger than those of the standard STIRAP $u_{\max}≃6/T$.

**Figure 2:**
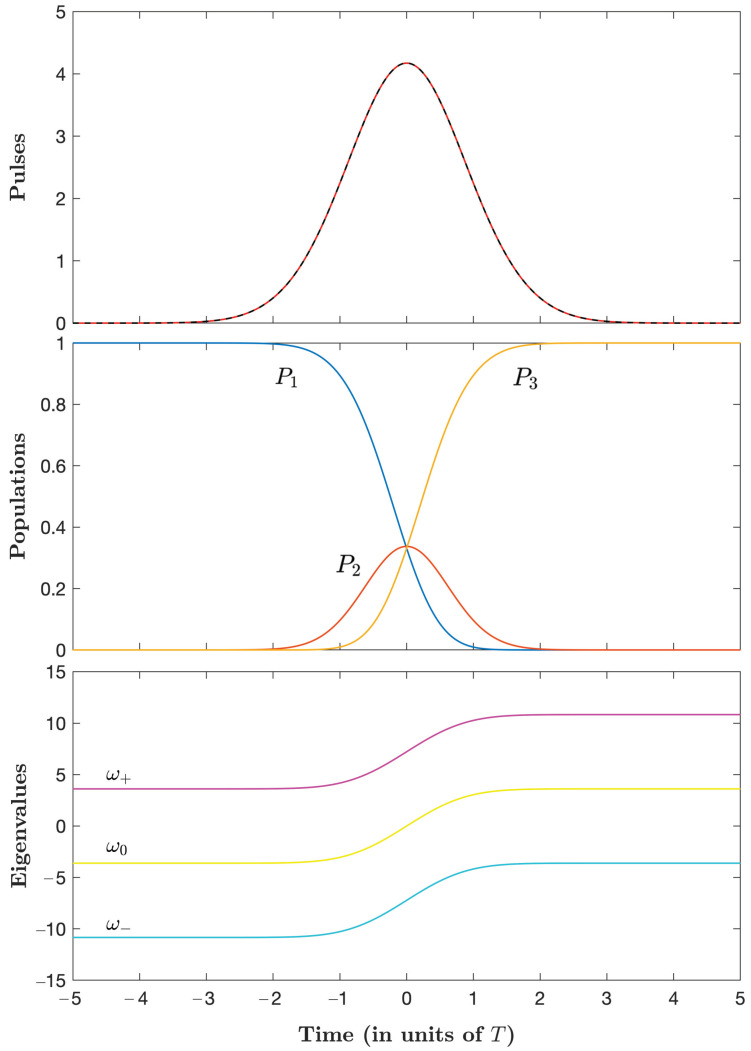
Parallel STIRAP dynamics for $\Omega_{0}=14.453/T$. Upper frame: The coincident control pulses $u_{P}\left(t\right)$ and $u_{S}\left(t\right)$ (in units of 1/T) according to (A44). Middle frame: Populations. Lower frame: The eigenvalues given by (A38) (in units of 1/T).

We next compare the energy-optimal dissipative STIREP with respect to Stimulated Raman parallel adiabatic passage (parallel STIRAP) with coincident pulses [28], where non-adiabatic transfer is ideally cancelled (in the adiabatic limit) [29], see Appendix D. Numerical implementations are shown in Figure 2 for $\Omega_{0}=14.453/T$, in a situation giving the same energy of the pulses as that for energy-optimal STIREP $E≃56\frac{\hbar }{T}$. We obtain the time area of the transient population in the excited state A≃0.535T, much larger than for energy-optimal STIREP A≃0.036T. Comparing the pulses, we first notice that the peak in parallel STIRAP $u_{\max}≃4.2/T$ is roughly five times smaller than the one in energy-optimal STIREP $u_{\max}≃19.6/T$. The duration of the control process is greater than for energy-optimal STIREP.

### 3.5. Analogy with a Planar Pendulum

We can reformulate the optimization problem in the limit of fully overlapping equal control fields:

(40) $$\begin{array}{c} u_{p}\left(t\right)=u_{s}\left(t\right)\equiv u\left(t\right), \end{array}$$

and show that it becomes analogous to the planar pendulum tipping over from the initial unstable vertical position.

We denote by a(t) the area at time *t* of u(t):

(41) $$\begin{array}{c} a\left(t\right)=\int_{0}^{t}u\left(t^{\prime }\right)dt^{\prime }. \end{array}$$

In this case, the dynamics can be exactly integrated:

(42) $$\begin{array}{c} x\left(t\right)={\left[\frac{1+\cos(\sqrt{2}a)}{2},\;\frac{\sin(\sqrt{2}a)}{\sqrt{2}},\;\frac{1-\cos(\sqrt{2}a)}{2}\right]}^{T}, \end{array}$$

showing that the target state is reached if:

(43) $$\begin{array}{c} a\left(T\right)=\frac{\pi }{\sqrt{2}}. \end{array}$$

Minimizing the energy of the controls (5) under the constraint of a given loss (7) is equivalent to finding the optimal solutions which minimize the energy $\int_{0}^{T}u{\left(t\right)}^{2}dt=\int_{0}^{T}{\dot{a}}^{2}dt$ while satisfying the constraint condition of an admissible given loss $2A=\int_{0}^{T}\sin^{2}\left(\sqrt{2}a\right)dt$. For that purpose, we introduce the Lagrangian equation:

(44) $$\begin{array}{c} L_{\lambda }={\dot{a}}^{2}+\lambda \sin^{2}\left(\sqrt{2}a\right), \end{array}$$

where λ is a Lagrange multiplier, aiming at minimizing $\int_{0}^{T}L_{\lambda }\left(t\right)dt$. A necessary condition is given by the Euler–Lagrange principle:

(45) $$\frac{\partial L_{\lambda }}{\partial a}-\frac{d}{dt}\left(\frac{\partial L_{\lambda }}{\partial \dot{a}}\right)=0,$$

giving the equation of a planar pendulum:

(46) $$\begin{array}{c} \ddot{a}-\frac{\lambda }{\sqrt{2}}\sin\left(2\sqrt{2}a\right)=0 \end{array}$$

where λ is directly connected to the frequency of the pendulum, *a* (up to a factor) is the angle (where 0 corresponds to the unstable vertical positon), and u(t) is the angular velocity. This equation can be integrated by using Jacobi functions with the boundary conditions a(0)=0 and $a\left(T\right)=\pi /\sqrt{2}$:

(47) $$\begin{array}{c} \dot{a}=u\left(t\right)=\sqrt{-\frac{\lambda }{m}}\sqrt{1-m\sin^{2}\left(\sqrt{2}a\right)} \end{array}$$

with *m* a constant related to the initial velocity u(0) and λ:

(48) $$\begin{array}{c} \dot{a}\left(0\right)=u\left(0\right)=\sqrt{-\frac{\lambda }{m}}. \end{array}$$

We derive, using $b=\sqrt{2}a$:

(49) $$\begin{array}{c} \int_{0}^{\sqrt{2}a}\frac{db}{\sqrt{1-m\sin^{2}b}}\equiv F\left(\sqrt{2}a|m\right)=\sqrt{-\frac{2\lambda }{m}}\;t, \end{array}$$

and

(50) $$\begin{array}{c} \int_{0}^{\pi /2}\frac{db}{\sqrt{1-m\sin^{2}b}}\equiv K\left(m\right)=\sqrt{-\frac{2\lambda }{m}}\frac{T}{2}, \end{array}$$

giving the relation between λ, *m* and *T*, i.e., by inverting (49):

(51) $$\begin{array}{c} \mathrm{sn}\left(F(\sqrt{2}a|m)\right)=\sin\left(\sqrt{2}a\right)=\mathrm{sn}\left(\frac{2K(m)t}{T},m\right) \end{array}$$

and

(52) $$\begin{array}{c} u\left(t\right)=\frac{\sqrt{2}K\left(m\right)}{T}\sqrt{1-m\;{\mathrm{sn}}^{2}\left(\frac{2K(m)t}{T},m\right)}. \end{array}$$

One can notice that the initial velocity $u\left(0\right)=\sqrt{2}K\left(m\right)/T$ is strictly positive, which induces the pendulum to tip over. In the limit of large negative *m*, this initial velocity goes to zero and we recover the pulse shape (34):

(53) $$\begin{array}{c} u\left(t\right)⇝\frac{\sqrt{-2m}K\left(m\right)}{T}\;\;\mathrm{sn}\left(\frac{2K(m)t}{T},m\right). \end{array}$$

## 4. Time-Optimal Dissipative STIREP

### 4.1. Construction of the Pseudo-Hamiltonian

In the time-optimal control problem, we minimize the following equation:

(54) $$J=\int_{0}^{T}dt,$$

where *T* is the control duration of the process to be determined optimally, under the constraint on the total peak amplitude of the fields, bounded by a constant $u_{0}$:

(55) $$\begin{array}{c} \sqrt{u_{1}^{2}\left(t\right)+u_{2}^{2}\left(t\right)}\leq u_{0}. \end{array}$$

The pesudo-Hamiltonian, which includes the subtraction of the integral kernel of the above cost, i.e., the constant $-p_{0}$, to which we add $p_{0}$, reads:

(56) $$\begin{array}{c} H_{c}\;=u_{p}\left(\lambda_{2}x_{1}-\lambda_{1}x_{2}\right)+u_{s}\left(\lambda_{3}x_{2}-\lambda_{2}x_{3}\right)+\mu x_{2}^{2}, \end{array}$$

where the costate Λ has four components $\Lambda ={\left[\lambda^{T},\mu \right]}^{T}$ with $\lambda ={\left[\lambda_{1},\lambda_{2},\lambda_{3}\right]}^{T}$. The adjoint equations of the costate are as follows:

(57) $$\begin{array}{cc} {\dot{\lambda }}_{1} & =-\frac{\partial H_{c}}{\partial x_{1}}=-\lambda_{2}u_{p}, \end{array}$$

(58) $$\begin{array}{cc} {\dot{\lambda }}_{2} & =-\frac{\partial H_{c}}{\partial x_{2}}=\lambda_{1}u_{p}-\lambda_{3}u_{s}-2\mu x_{2}, \end{array}$$

(59) $$\begin{array}{cc} {\dot{\lambda }}_{3} & =-\frac{\partial H_{c}}{\partial x_{3}}=\lambda_{2}u_{s}, \end{array}$$

(60) $$\begin{array}{cc} \dot{\mu } & =0\;i.e.,\;\mu =const. \end{array}$$

The pseudo-Hamiltonian is of the form (A45) $H_{c}=H_{0}+u_{p}H_{p}+u_{s}H_{s}$ (see Appendix E), with the control variables $u_{p}$ and $u_{s}$, and $H_{p}=\lambda_{2}x_{1}-\lambda_{1}x_{2}$, $H_{s}=\lambda_{3}x_{2}-\lambda_{2}x_{3}$, we can, thus, apply the results of Appendix E:

(61) $$\begin{array}{c} u_{p}=\left(\lambda_{2}x_{1}-\lambda_{1}x_{2}\right)/R,\;u_{s}=\left(\lambda_{3}x_{2}-\lambda_{2}x_{3}\right)/R \end{array}$$

with

(62) $$R=\sqrt{{\left(\lambda_{2}x_{1}-\lambda_{1}x_{2}\right)}^{2}+{\left(\lambda_{3}x_{2}-\lambda_{2}x_{3}\right)}^{2}}.$$

This leads to:

(63) $$H_{c}=R+\mu x_{2}^{2},$$

and the controls attain the maximum of the constraint at each time:

(64) $$\begin{array}{c} u_{p}^{2}+u_{s}^{2}=u_{0}^{2}. \end{array}$$

We can make a change of variables for the time, renormalizing the field amplitude as follows:

(65) $$\begin{array}{cc} {\tilde{u}}_{p} & =\frac{u_{p}}{\sqrt{u_{p}^{2}+u_{s}^{2}}}=\frac{u_{p}}{u_{0}}, \end{array}$$

(66) $$\begin{array}{cc} {\tilde{u}}_{s} & =\frac{u_{s}}{\sqrt{u_{p}^{2}+u_{s}^{2}}}=\frac{u_{s}}{u_{0}}, \end{array}$$

(67) $$\begin{array}{cc} \tilde{t} & =u_{0}t, \end{array}$$

i.e.,

(68) $$\begin{array}{c} {\tilde{u}}_{p}^{2}+{\tilde{u}}_{s}^{2}=1, \end{array}$$

and the equation becomes:

(69) $$\begin{array}{c} \frac{d}{d\tilde{t}}|\tilde{x}\rangle =\tilde{A}|\tilde{x}\rangle ,\;|\tilde{x}\left(\tilde{t}\right)\rangle \equiv |x\left(t\right)\rangle ,\;\tilde{A}=\left[\begin{array}{ccc} 0 & -{\tilde{u}}_{p} & 0\\ {\tilde{u}}_{p} & 0 & -{\tilde{u}}_{s}\\ 0 & {\tilde{u}}_{s} & 0 \end{array}\right]. \end{array}$$

This means that we can always renormalize the field amplitudes by modifying the optimal time accordingly. We will consider below the tilde variables (corresponding to finding the optimal time for $u_{0}=1$).

Introducing the following angle coordinates:

(70) $$\begin{array}{c} x_{1}=\cos\varphi \cos\theta ,\;x_{2}=\sin\varphi ,\;x_{3}=\cos\varphi \sin\theta \end{array}$$

with the initial condition $\varphi (t_{i})=0$, $\theta (t_{i})=0$ and the final condition $\varphi (t_{f})=0$, $\theta (t_{f})=\pi /2$. The equations of the dynamics (69) can be simplified as follows:

(71) $$\begin{array}{c} \dot{\varphi }={\tilde{v}}_{p},\;\dot{\theta }=-{\tilde{v}}_{s}\tan\varphi , \end{array}$$

after a rotation on the control fields:

(72) $$\left[\begin{array}{c} {\tilde{v}}_{p}\\ {\tilde{v}}_{s} \end{array}\right]=\left[\begin{array}{cc} \cos\theta & -\sin\theta \\ -\sin\theta & -\cos\theta \end{array}\right]\left[\begin{array}{c} {\tilde{u}}_{p}\\ {\tilde{u}}_{s} \end{array}\right],$$

leading to an invariant cost on the new field variables since ${\tilde{u}}_{p}^{2}+{\tilde{u}}_{s}^{2}={\tilde{v}}_{p}^{2}+{\tilde{v}}_{s}^{2}=1$. We arrive at:

(73) $$\begin{array}{c} H_{c}-\mu \sin^{2}\varphi =\lambda_{\varphi }\;\dot{\varphi }+\lambda_{\theta }\;\dot{\theta }=\lambda_{\varphi }\;{\tilde{v}}_{p}-\lambda_{\theta }\;{\tilde{v}}_{s}\tan\varphi \end{array}$$

with $\Lambda ={\left[\lambda_{\varphi },\lambda_{\theta },\mu \right]}^{T}$ being the costate gathering the conjugate momenta of φ, θ, and *y*, respectively. The following Hamilton Equations:

(74) $$\begin{array}{c} \dot{\varphi }=\frac{\partial H_{c}}{\partial \lambda_{\varphi }},\;\dot{\theta }=\frac{\partial H_{c}}{\partial \lambda_{\theta }}, \end{array}$$

lead to the equations of motion (71) and to:

(75) $$\begin{array}{cc} {\dot{\lambda }}_{\varphi } & =-\frac{\partial H_{c}}{\partial \varphi }=\frac{{\tilde{v}}_{s}}{\cos^{2}\varphi }\lambda_{\theta }-\mu \sin\left(2\varphi \right), \end{array}$$

(76) $$\begin{array}{cc} {\dot{\lambda }}_{\theta } & =-\frac{\partial H_{c}}{\partial \theta }=0, \end{array}$$

(77) $$\begin{array}{cc} \;\dot{\mu } & =-\frac{\partial H_{c}}{\partial y}=0. \end{array}$$

This implies that $\lambda_{\theta }$ and μ are constants of motion. $H_{c}$ (73) is again of the form (A45), implying:

(78) $$H_{c}=\sqrt{\lambda_{\varphi }^{2}+\lambda_{\theta }^{2}\tan^{2}\varphi }+\mu \sin^{2}\varphi ,$$

and

(79) $${\tilde{v}}_{p}=\frac{\lambda_{\varphi }}{R},\;{\tilde{v}}_{s}=-\frac{\lambda_{\theta }\tan\varphi }{R},$$

with

(80) $$\begin{array}{c} R=\sqrt{\lambda_{\varphi }^{2}+\lambda_{\theta }^{2}\tan^{2}\varphi }. \end{array}$$

The equations of motion read then:

(81) $$\begin{array}{cc} \dot{\varphi } & =\frac{\lambda_{\varphi }}{R}, \end{array}$$

(82) $$\begin{array}{cc} \dot{\theta } & =\frac{\lambda_{\theta }\tan^{2}\varphi }{R}, \end{array}$$

(83) $$\begin{array}{cc} \dot{y} & =\sin^{2}\varphi , \end{array}$$

(84) $$\begin{array}{cc} {\dot{\lambda }}_{\varphi } & =-\lambda_{\theta }^{2}\frac{\sin\varphi }{R\cos^{3}\varphi }-\mu \sin\left(2\varphi \right) \end{array}$$

with the boundary conditions:

(85) $$\begin{array}{cc} \varphi \left(t_{i}\right)=0,\;\theta \left(t_{i}\right) & =0,\;y(t_{i})=0, \end{array}$$

(86) $$\begin{array}{cc} \varphi \left(t_{f}\right)=0,\;\theta \left(t_{f}\right) & =\pi /2,\;y(t_{f})=A. \end{array}$$

### 4.2. Construction of the Optimal Trajectories from the PMP

Since $H_{c}=h$ is a constant, we obtain:

(87) $$\lambda_{\varphi }=\pm \sqrt{{\left(h-\mu \sin^{2}\varphi \right)}^{2}-\lambda_{\theta }^{2}\tan^{2}\varphi }.$$

Following the same lines as in the energy minimum case, we have:

(88) $$\begin{array}{cc} \dot{\varphi } & =\pm \frac{\sqrt{{\left(1-\tilde{\mu }\sin^{2}\varphi \right)}^{2}-{\tilde{\lambda }}_{\theta }^{2}\tan^{2}\varphi }}{1-\tilde{\mu }\sin^{2}\varphi }, \end{array}$$

(89) $$\begin{array}{cc} \frac{d\varphi }{d\theta } & =\pm \frac{\sqrt{{\left(1-\tilde{\mu }\sin^{2}\varphi \right)}^{2}-{\tilde{\lambda }}_{\theta }^{2}\tan^{2}\varphi }}{{\tilde{\lambda }}_{\theta }\tan^{2}\varphi } \end{array}$$

with the normalized constants:

(90) $$\begin{array}{c} \tilde{\mu }=\frac{\mu }{h},\;{\tilde{\lambda }}_{\theta }=\frac{\lambda_{\theta }}{h}. \end{array}$$

We know that φ has by symmetry to reach its maximum value at t=T/2: $\dot{\varphi }\left(T/2\right)=0$, i.e.,

(91) $${\left(1-\tilde{\mu }\sin^{2}\varphi_{0}\right)}^{2}={\tilde{\lambda }}_{\theta }^{2}\tan^{2}\varphi_{0}$$

with the notation $\varphi_{0}\equiv \varphi \left(T/2\right)$. This equation shows a dependence upon ${\tilde{\lambda }}_{\theta }^{2}$. We can, thus, limit our study to positive ${\tilde{\lambda }}_{\theta }$. It is solved in Appendix F.

We next solve the differential Equation (89) numerically and determine which values of $\tilde{\mu }$ and ${\tilde{\lambda }}_{\theta }$ satisfy the following:

(92) $$\varphi \left(\theta =\pi /4\right)=\varphi_{0},$$

where the left-hand side is the numerical solution of (89) and the right-hand side the possible solution(s) $\varphi_{0}$, as determined in Appendix F.

We note that for $\tilde{\mu }\leq -8$, (92) is satisfied only for the smallest root $\varphi_{0}$ [i.e., (A77) for k=1], since φ (assumed positive) grows from 0 to $\varphi_{0}$, where dφ/dθ=0. In addition, we emphasize that the results become strongly sensitive to ${\tilde{\lambda }}_{\theta }$ for large negative $\tilde{\mu }$, which necessitates a high precision on the estimation of the parameter. For $\tilde{\mu }\in ]-8,1]$, there is one root of $\varphi_{0}$ (A74), and (92) can be always satisfied. For $\tilde{\mu }>1$, no solution satisfying (92) exists. The couples ${\tilde{\lambda }}_{\theta },\tilde{\mu }$ and the corresponding $\varphi_{0}$ are shown in Table 2.

**Figure 3:**
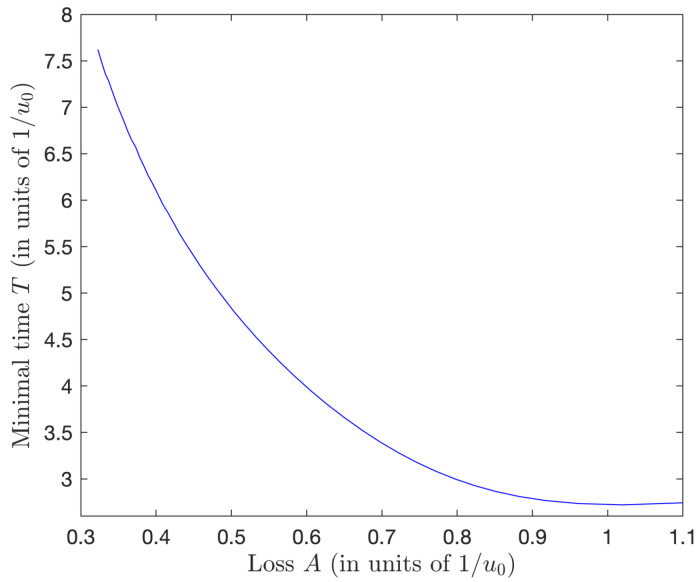
Minimal time as a function of the normalized loss from Table 2.

Using these values of the couple ($\tilde{\mu }$, ${\tilde{\lambda }}_{\theta }$), we can solve the differential Equation (88), to determine the value of $t_{0}$ when $\varphi \left(t_{0}\right)=\varphi_{0}$, and thus to obtain the optimal time $T=2t_{0}$, and the resulting normalized loss: $A=\int_{t_{i}}^{t_{f}}dt\;x_{2}{\left(t\right)}^{2}=2\int_{0}^{t_{0}}dt\;\sin^{2}\varphi \left(t\right)$. We show the corresponding data in Table 2, from which one can conclude that the optimal control time *T* decreases for increasing values of *A* with diminishing rates and gradually flattens out for large *A*. This is shown in Figure 3.

We observe from Table 2 that, for the unconstrained case, i.e., $\tilde{\mu }=0$, the determined time area is $A≃1.0203/u_{0}$, while the optimal time is $T=2.7207/u_{0}$, which recovers the unconstrained result obtained in the energy-optimal dissipative STIREP (see Table 1) [i.e., Equation (32)]: $A=\frac{3T}{8}≃1.0203/u_{0}$, and $\varphi_{0}=\pi /3\approx 1.0472$.

### 4.3. Derivation of the Pulses and the Dynamics

From a given pair $(\tilde{\mu }$, ${\tilde{\lambda }}_{\theta })$, φ(t) can be obtained numerically by solving the differential Equation (88), and then $\lambda_{\varphi }\left(t\right)$ is derived from (87). The original controls ${\tilde{u}}_{p}$, ${\tilde{u}}_{s}$ are obtained from Equation (72), where the angle θ is derived numerically from Equation (89):

(93) $$\begin{array}{c} \theta =\pm \int_{\varphi_{i}}^{\varphi }\frac{{\tilde{\lambda }}_{\theta }\tan^{2}\varphi }{\sqrt{{\left(1-\tilde{\mu }\sin^{2}\varphi \right)}^{2}-{\tilde{\lambda }}_{\theta }^{2}\tan^{2}\varphi }}d\varphi . \end{array}$$

**Figure 4:**
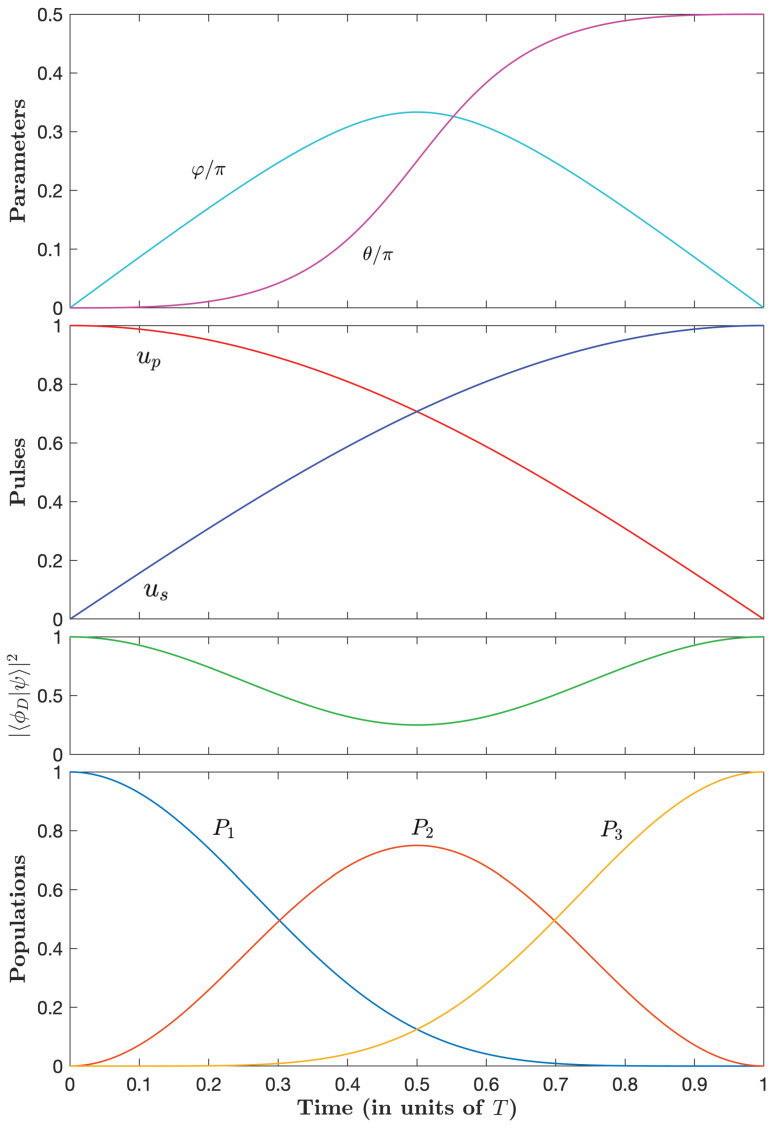
Time dependence of the parameters φ and θ (upper frame); the control pulse amplitudes $u_{p}$ and $u_{s}$ (in units of $u_{0}$) (upper middle frame); the projection (in absolute value squared) of the dynamics onto the dark state (94) (lower middle frame); and populations (lower frame) for the case $\tilde{\mu }=0$ corresponding to unconstrained optimal pulses with the normalized loss $A\approx 1.02/u_{0}$ and the optimal time $T\approx 2.72/u_{0}$.

**Figure 5:**
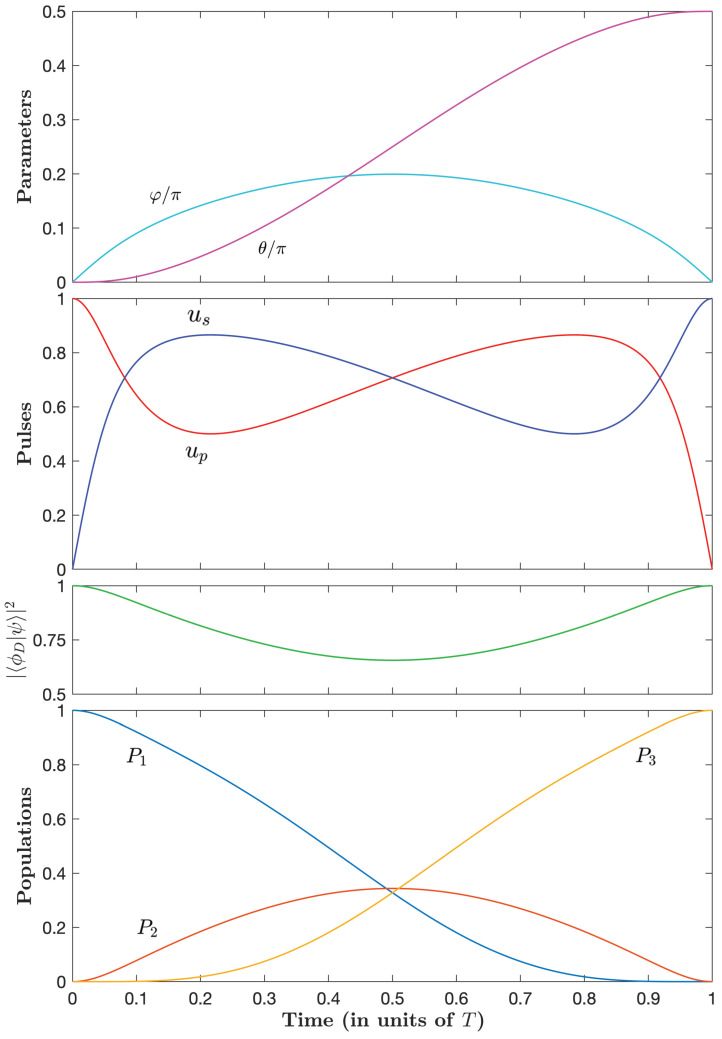
Same as Figure 4, but for $\tilde{\mu }=-5$, i.e., $A\approx 0.7/u_{0}$, and $T\approx 3.98/u_{0}$.

**Figure 6:**
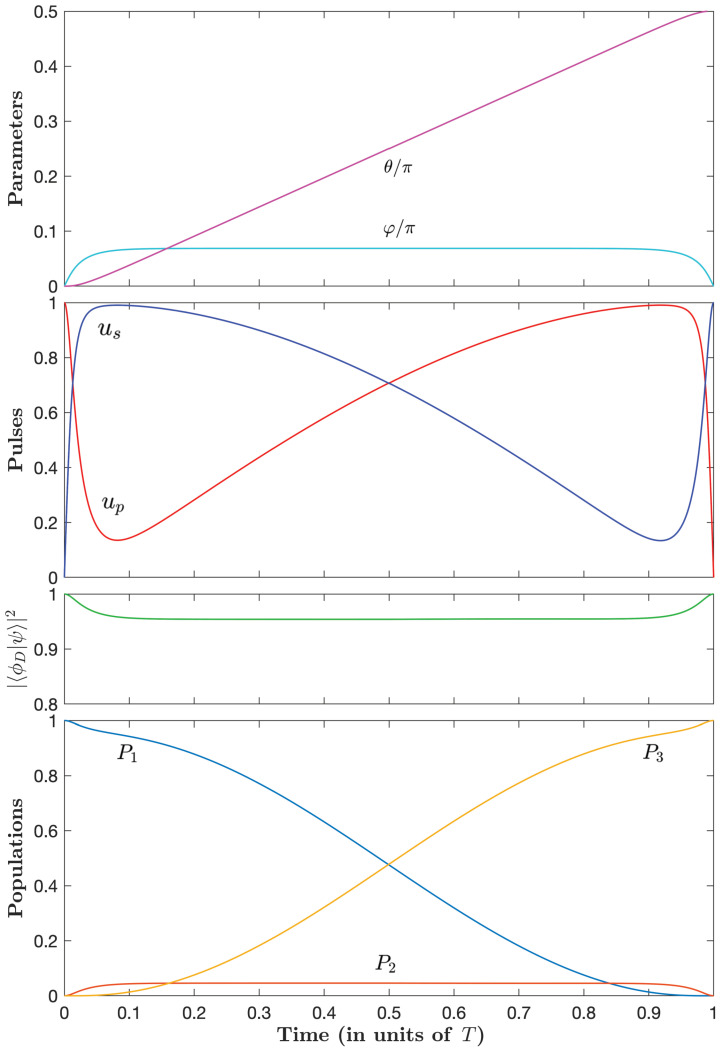
Same as Figure 4, but for $\tilde{\mu }=-24$, i.e., $A\approx 0.32/u_{0}$, and $T\approx 7.62/u_{0}$.

Figure 4, Figure 5 and Figure 6 show the parameters φ(t) and θ(t) and the dynamics for three typical couples of ($\tilde{\mu }$, ${\tilde{\lambda }}_{\theta }$) with decreasing losses.

Starting with the full intuitive dynamics in the unconstrained case (Figure 4), we observe that, as the optimal time increases (i.e., $\tilde{\mu }$ becomes larger in absolute value and negative), the pump pulse decreases sharply at early times before slowly increasing and the Stokes pulse increases sharply at early times before slowly decreasing (and a symmetric situation at late times). This corresponds to a slow counterintuitive sequence, reminiscent of the STIRAP sequence, sandwiched by two fast intuitive sequences. This remarkable simple optimal sequence, referred to as an intuitive/counterintuitive/intuitive (ICI) sequence, represents an important finding of our paper. Figure 4, Figure 5 and Figure 6 show that this behavior is more pronounced, i.e., with a sharper intuitive sequence with higher peak amplitude, for a decreasing admissible loss.

The projection of the dynamics onto a dark state (i.e., having no component in the excited state):

(94) $$\begin{array}{cc} |\phi_{D}\left(t\right)\rangle & =\left[\begin{array}{c} \cos\theta \\ 0\\ -\sin\theta \end{array}\right], \end{array}$$

which we define with the actual θ, gets closer to one for a smaller admissible loss. We can notice that this projection is in fact 1 minus the population in state |2〉.

## 5. Conclusions and Discussion

In this paper, we have derived energy- and time-minimum optimizations under the constraint of a given admissible loss leading to exact state-to-state transfer of three-level Λ-type quantum systems. The resulting control fields have very different shapes depending on the considered optimization.

In the case of energy-optimal dissipative STIREP, the pulses feature an intuitive sequence similar to the unconstrained situation, of amplitudes increasing with the decrease of the given loss, tending to coincident pulses below the loss A≲0.08T. We obtain in this case the analytic expressions of the pulses, as a sine Jacobi elliptic function. The energy-optimal dynamics operates with a relatively strong field in the limit of a low loss, and far from a dark state.

In the case of time-optimal dissipative STIREP, the control time increases with the decrease of the given loss *A*. The process features a remarkable pulse sequence: a relatively slow counterintuitive sequence sandwiched by sharp intuitive sequences (see Figure 6) and the ICI sequence, sharing, thus, some similarities with STIRAP except the very beginning and the very end of the process, which are strongly non-adiabatic (see below for a detailed comparison). The time-optimal strategy operates relatively close to the dark state in the limit of a low admissible loss.

**Figure 7:**
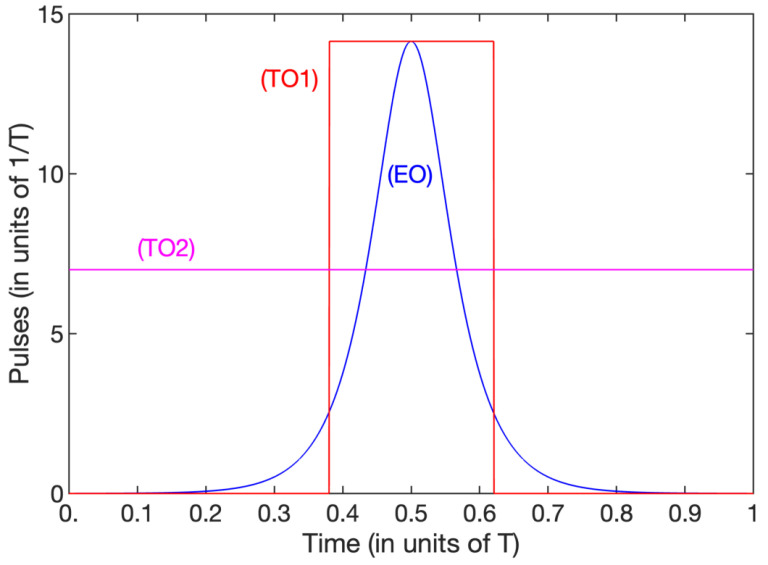
Pulse amplitudes for the three examples, i.e., EO, TO1, TO2, described in the text. For TO1 and TO2, we have only plotted the respective peak amplitudes $u_{0,\mathrm{TO}1}$, $u_{0,\mathrm{TO}2}\approx 7/T$ as lines, the pulse shapes being of the form in Figure 5 and Figure 6, respectively.

The process duration, peak pulse amplitude, energy, and area for several given admissible losses are reported in Table 1 and Table 2. One can compare a few values in order to highlight the main features and differences of the two optimization strategies:

- The energy optimization with the (low) given admissible loss A=0.05T with $T\equiv T_{\mathrm{EO}}$ the time of the process, referred to as EO, yields $A\approx 0.7/u_{\max,\mathrm{EO}}$ with $u_{\max,\mathrm{EO}}$ the peak of the pulse, $T_{\mathrm{EO}}\approx 14/u_{\max,\mathrm{EO}}$, the energy $E_{\mathrm{EO}}=40\hbar /T_{\mathrm{EO}}\approx 2.8u_{\max,\mathrm{EO}}$, and the pulse area $A_{\mathrm{EO}}=\pi$;
- The time-optimization with the same loss A=0.05T and the same peak amplitude $u_{0,\mathrm{TO}1}\equiv u_{\max,\mathrm{EO}}$, referred to as TO1, is roughly obtained for $\tilde{\mu }=-5$: $A\approx 0.7/u_{0,\mathrm{TO}1}$, $T_{\mathrm{TO}1}\approx 3.4/u_{0,\mathrm{TO}1}$, $E_{\mathrm{TO}1}\approx 11.5\hbar /T_{\mathrm{TO}1}\approx 3.4u_{0}$ and $A_{\mathrm{TO}1}=3.4$; it shows a much smaller (roughly four times smaller) time of processing, but slightly larger pulse area and energy;
- The time-optimization with the same loss A=0.05T and the same duration as the energy optimization: $T_{\mathrm{TO}2}\equiv T\equiv T_{\mathrm{EO}}$, referred to as TO2, is roughly obtained for $\tilde{\mu }=-20.5$: this leads to a significant larger energy $E_{\mathrm{TO}2}=49\hbar /T$ and a twice larger pulse area $A_{\mathrm{TO}2}=7$, but to a (twice) smaller peak pulse amplitude $u_{0,\mathrm{TO}2}\approx 7/T$.
  We show the dependence of the pulse amplitude on the duration corresponding to these three examples in Figure 7. The energy-minimization strategy can, thus, achieve the transfer for a given loss in a relatively small pulse area, but with a relatively large pulse peak amplitude due to its sharp shape. On the other hand, the time-minimization strategy can achieve it with a weaker pulse amplitude, but for a larger pulse area (and energy).

**Figure 8:**
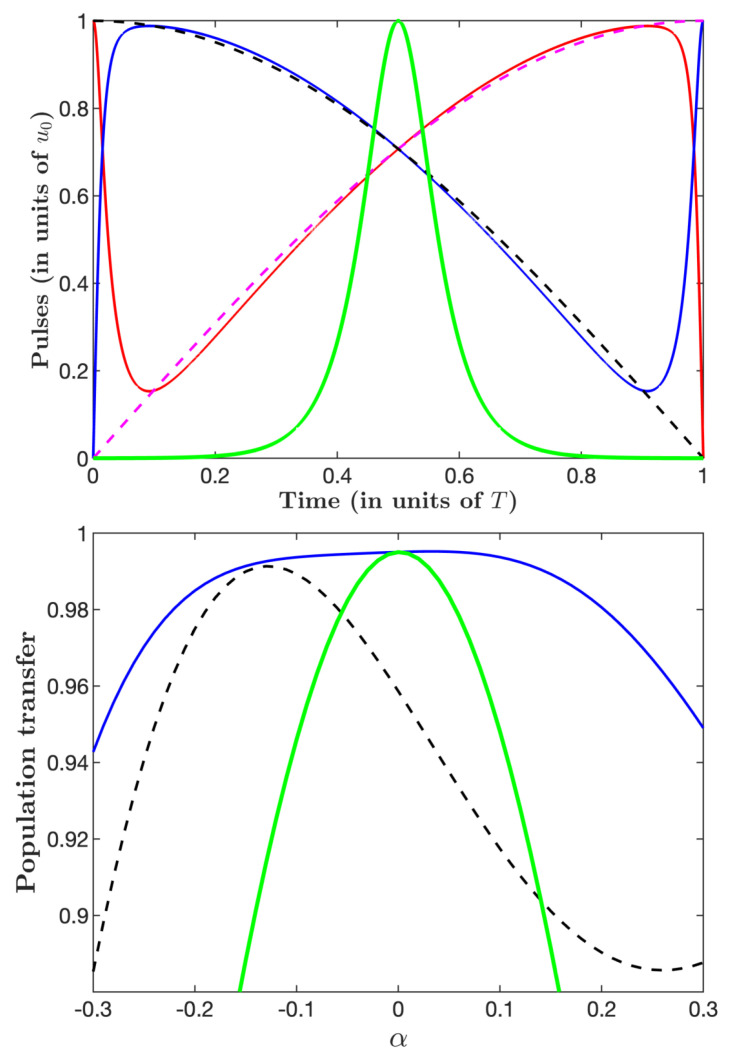
Robustness profiles (lower frame) of the the energy-optimal (EO) (thick lines), time-optimal (TO2) (full lines), and associated STIRAP (dashed lines) pulses (upper frame) for the duration $T=7/u_{0}$ and the loss A=0.05T and Γ=0.1/T, i.e., $P_{\mathrm{loss}}\approx 5\times 10^{-3}$.

In Figure 8, we study the robustness as a function of a relative deviation α of the amplitudes, i.e., with the amplitudes $u_{j}\left(1+\alpha \right)$, j=p,s, taking into account explicitly the loss, i.e., using the Hamiltonian Equation (1). We consider the three cases: energy optimization with A=0.05T (EO), time optimization (TO2), and the associated STIRAP. The latter is defined as the traditional counterintuitive configuration of the pumps and Stokes pulses with sine and cosine shapes, respectively, that fit well the actual TO2 pulses except the initial and final sharp intuitive sequences (see the upper frame of Figure 8). We observe that the time-optimal ICI sequence TO2 features a flat asymmetric profile on a relatively large zone, and that it is much more robust than its associated STIRAP. The lack of robustness of the latter is expected, as the total pulse area is too low to achieve efficient adiabaticity.

Comparing with the energy-optimal dissipative STIREP, the time-optimal ICI sequence is much more robust. The weak robustness of the energy-minimization pulse sequence can be understood by the fact that this process can be interpreted as the counterpart of the two-state π-pulse transfer to the Λ system with a large amplitude and a short time in order to respect the admissible loss.

We conclude that the time-optimal ICI sequence achieves precise and fast population transfer, with a chosen loss, and in a robust way (in the limit of low loss).

We notice that the time-optimal ICI sequence is very similar to the pulse sequence derived in [15] (compare Figure 6 with Figure 3 of Ref. [15]),where the optimization was determined with the explicit constraint of robustness, but without consideration of loss.

The implementation of the ICI sequence with a high fidelity of error $P_{\mathrm{loss}}≲10^{-3}$ ($P_{\mathrm{loss}}\approx 10^{-3}$ is the situation considered in Figure 8) corresponds to $T≲2\times 10^{-2}/\Gamma$ with the typical value A=0.05T and $u_{0}=7/T$, i.e., $u_{0}≳350\Gamma$.

For a practical implementation, we have to consider the additional decay within the Λ system, including decay channels from state |e〉 to the two ground states |1〉 and |3〉 and the associated decoherence, which has to be analyzed with the density matrix formulation and the Lindblad equation, as detailed in Appendix G. Numerical analysis has been conducted for the time-optimal situation, which is the situation of interest since it features robustness and low loss for large enough $\tilde{\mu }$. We obtain that the two additional channels |e〉→|1〉 and |e〉→|3〉 (associated with the respective rates $\gamma_{1}$ and $\gamma_{3}$) add each a contribution of half the external loss given by the Γ-channel (for the same rate). We obtain more specifically for the total loss:

(95) $$P_{\mathrm{loss}}\approx P_{\mathrm{loss}}^{\left(\Gamma \right)}\left(1+\frac{\gamma_{1}}{2\Gamma }+\frac{\gamma_{3}}{2\Gamma }\right),$$

with the external loss given by the Γ-channel defined by (2): $P_{\mathrm{loss}}^{\left(\Gamma \right)}=\Gamma \int_{t_{i}}^{t_{f}}dt{\left|c_{2}\right|}^{2}$. In practice, we have this to consider the total loss $P_{\mathrm{loss}}$ (95).

Concerning a possible experimental implementation, one can mention the excited state ${}^{1}$D${}_{2}$ of a praseodymium ion in a Pr${}^{3+}$:Y${}_{2}$SiO${}_{5}$ crystal with $\Gamma^{-1}\approx 164\;\mu$s [30,31], which requires the Rabi frequency $u_{0}≳2\pi \times 340$ kHz and the duration T≲3.3μs, well in the achievable experimental range.

## Figures and Tables

**Table 1:** Energy-optimal parameters for various normalized losses *A*: optimal values of *m*, $\varphi_{0}$, and the corresponding energy E, peak value $u_{\max}$, and pulse area $A=\int_{0}^{T}ds\sqrt{u_{p}^{2}+u_{s}^{2}}$. The pulse area for the Rabi frequency is 2A.

*A* (in units of *T*)	3/8	0.35	0.3	0.2	0.15
*m*	0	−0.264	−1.123	−9.4	−49.97
$\varphi_{0}$	π/3	1.0140	0.9502	0.8364	0.7995
E (in units of 1/T)	7.40	7.44	7.79	10.28	13.40
$u_{\max}$ (in units of 1/T)	2.72	2.51	2.54	3.60	4.73
A	2.72	2.72	2.75	2.91	3.03
*A* (in units of *T*)	0.1	0.08	0.05	0.04	0.03
*m*	−1379	−16770	$-3.032\times 10^{7}$	$-4.5\times 10^{9}$	$-1.87\times 10^{13}$
$\varphi_{0}$	0.7862	0.7855	0.7854	0.7854	0.7854
E (in units of 1/T)	20.00	25.00	40.00	50.00	66.66
$u_{\max}$ (in units of 1/T)	7.07	8.84	14.14	17.68	23.57
A	3.12	3.14	3.14	3.14	3.14

**Table 2:** Time-optimal $\varphi_{0}$ as a function of the couple ${\tilde{\lambda }}_{\theta }$, $\tilde{\mu }$ satisfying (92), and the corresponding normalized loss *A*, minimal time *T*, ratio A/T, pulse area A, and energy E. The values $\tilde{\mu }$ are given exactly and all the others quantities are approximate.

$\tilde{\mu }$	${\tilde{\lambda }}_{\theta }$	$\varphi_{0}$	*A* ($1/u_{0}$)	*T* ($1/u_{0}$)	A/T	A	E (ℏ/T)
0.5	0.25879	1.1527	1.1177	2.7477	0.407	2.75	7.54
0	0.57735	1.0472	1.0203	2.7207	0.375	2.72	7.40
−0.5	0.90476	0.9783	0.9612	2.7346	0.352	2.73	7.48
−1	1.23456	0.9248	0.9176	2.7668	0.332	2.77	7.65
−1.5	1.56434	0.8795	0.8821	2.8109	0.314	2.81	7.90
−2	1.89263	0.8389	0.8513	2.8647	0.297	2.86	8.21
−2.5	2.21825	0.8012	0.8234	2.9275	0.281	2.93	8.57
−3	2.53997	0.7652	0.7972	2.9994	0.266	3.00	9.00
−3.5	2.85642	0.7301	0.7720	3.0812	0.251	3.08	9.49
−4	3.16595	0.6955	0.7473	3.1739	0.236	3.17	10.07
−4.5	3.46665	0.6609	0.7228	3.2779	0.221	3.28	10.74
−5	3.75639	0.6266	0.6984	3.3938	0.206	3.39	11.52
−5.5	4.03322	0.5926	0.6742	3.5211	0.192	3.52	12.40
−6	4.29565	0.5597	0.6504	3.6580	0.178	3.66	13.38
−6.5	4.54311	0.5283	0.6273	3.8018	0.165	3.80	14.45
−7	4.77607	0.4992	0.6055	3.9494	0.153	3.95	15.60
−7.5	4.99577	0.4727	0.5850	4.0979	0.143	4.10	16.79
−8	5.20381	0.4489	0.5660	4.2449	0.133	4.24	18.02
−8.5	5.40180	0.4277	0.5485	4.3893	0.125	4.39	19.27
−9	5.59116	0.4088	0.5323	4.5296	0.118	4.53	20.52
−9.5	5.77306	0.3920	0.5175	4.6666	0.111	4.67	21.78
−10	5.94845	0.3770	0.5037	4.7988	0.105	4.80	23.03
−10.5	6.11800	0.3635	0.4913	4.9299	0.100	4.93	24.30
−11	6.28261	0.3514	0.4794	5.0551	0.095	5.06	25.55
−11.5	6.44250	0.3405	0.4684	5.1768	0.091	5.18	26.80
−12	6.59818	0.3305	0.4582	5.2963	0.087	5.30	28.05
−12.5	6.75000	0.3214	0.4488	5.4148	0.083	5.41	29.32
−13	6.89827	0.3130	0.4397	5.5278	0.080	5.53	30.56
−13.5	7.04324	0.3053	0.4311	5.6386	0.077	5.64	31.79
−14	7.18513	0.2981	0.4235	5.7525	0.074	5.75	33.09
−14.5	7.32416	0.2915	0.4160	5.8600	0.071	5.86	34.34
−15	7.46049	0.2853	0.4085	5.9613	0.069	5.96	35.54
−15.5	7.59429	0.2795	0.4020	6.0690	0.066	6.07	36.83
−16	7.72569	0.2740	0.3957	6.1722	0.064	6.17	38.10
−16.5	7.85483	0.2689	0.3892	6.2680	0.062	6.27	39.29
−17	7.98182	0.2640	0.3836	6.3699	0.060	6.37	40.58
−17.5	8.10678	0.2594	0.3779	6.4657	0.058	6.47	41.80
−18	8.22979	0.2551	0.3730	6.5687	0.057	6.57	43.15
−18.5	8.35096	0.2509	0.3671	6.6517	0.055	6.65	44.25
−19	8.47035	0.2470	0.3622	6.7434	0.054	6.74	45.47
−19.5	8.58806	0.2432	0.3577	6.8391	0.052	6.84	46.77
−20	8.70414	0.2397	0.3531	6.9296	0.051	6.93	48.02
−20.5	8.81868	0.2362	0.3486	7.0162	0.050	7.02	49.23
−21	8.93172	0.2330	0.3446	7.1074	0.049	7.11	50.51
−21.5	9.04333	0.2298	0.3406	7.1965	0.047	7.20	51.79
−22	9.15356	0.2268	0.3368	7.2843	0.046	7.28	53.06
−22.5	9.26246	0.2239	0.3325	7.3588	0.045	7.36	54.15
−23	9.37008	0.2211	0.3290	7.4475	0.044	7.45	55.47
−23.5	9.47647	0.2184	0.3234	7.4864	0.043	7.49	56.05
−24	9.58166	0.2159	0.3225	7.6230	0.042	7.62	58.11

**Figure A1:**
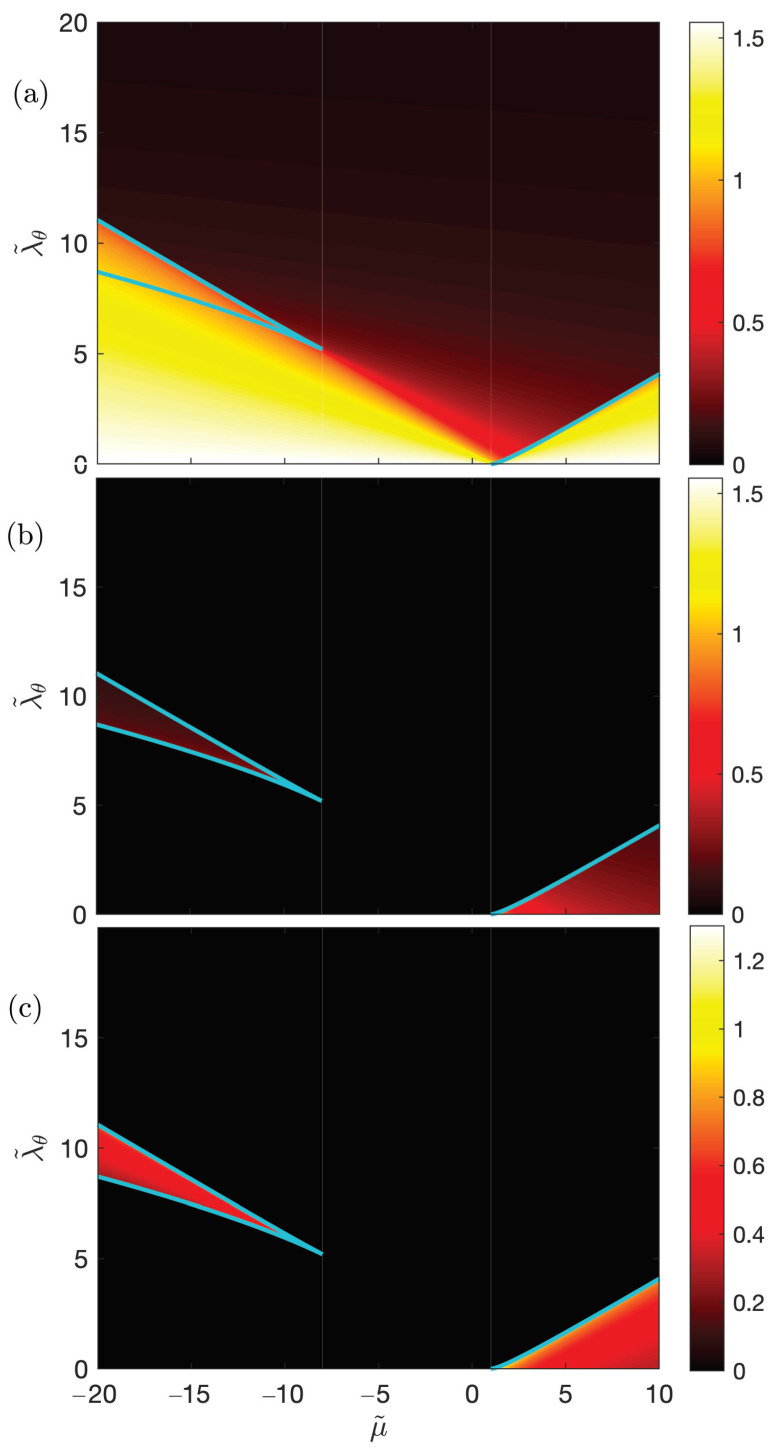
Contour plots of the solution(s) of $\varphi_{0}$ from Equation (91) as a function of $\tilde{\mu }$ and ${\tilde{\lambda }}_{\theta }$. Absence of solutions is indicated by black color (“0”). The situation of one solution is reported in (**a**) [i.e., the two others are set to zero in (**b**,**c**)]: the solution is localized outside the (blue) thick-line boundaries [corresponding to the roots (A73)]; the situation of two solutions is reported in (**a**,**b**): the solutions are localized on the (blue) thick-line boundaries; when there are three solutions, they are localized within the boundaries.

## Appendix A. Pontryagin’s Maximum Principle

To determine optimal control fields u(t) of a dynamical system $\dot{x}=f\left(x(t);u(t)\right)$ (of dimension *N*) with respect to the minimization of a given cost:

(A1) $$J\left(u(t)\right)=\int_{t_{i}}^{t_{f}}g\left(x(t),u(t)\right)dt,$$

Pontryagin’s maximum principle transforms the initial infinite-dimension control problem into a finite-dimension problem and allows discontinuous controls [18,20]. It provides necessary conditions for optimality. It states that the trajectories of the extremal vector x(t) and of the corresponding adjoint state p(t) formed by the Lagrange multipliers, $p\left(t\right)\equiv [p_{1}\left(t\right),\cdots ,p_{N}\left(t\right)]$, fulfill Hamilton’s equations:

(A2) $$\dot{x}=\frac{\partial H_{c}}{\partial p},\;\dot{p}=-\frac{\partial H_{c}}{\partial x},$$

associated with the control pseudo-Hamiltonian equation:

(A3) $$H_{c}\left(p(t),x(t);u(t)\right)=p\cdot f\left(x(t);u(t)\right)-p_{0}g\left(x(t),u(t)\right),$$

where the constant $p_{0}>0$ can be chosen for convenience since it amounts to multiply the cost function by a constant. For almost all $t_{i}\leq t\leq t_{f}$, the function $H_{c}\left(p(t),x(t);u(t)\right)$ is maximum at certain controls v(t)=u(t), for which one can write $H_{c}\left(p(t),x(t);v(t)\right)=const.$, i.e.,

(A4) $$\frac{\partial H_{c}}{\partial u}=0.$$

The costate λ defined via the conjugate moments: $\lambda =p^{T}$, is solution of the second Hamilton equation:

(A5) $${\dot{\lambda }}^{T}=-\frac{\partial H_{c}}{\partial x}=-\lambda^{T}\frac{\partial f}{\partial x}+p_{0}\frac{\partial g}{\partial x}.$$

## Appendix B. Integration of Equation (26)

In this Appendix, we solve the differential Equation (26):

(A6) $$\begin{array}{cc} \dot{\varphi } & =\lambda_{\varphi }=\pm \sqrt{2h-\sin^{2}\varphi \left(2\mu +\frac{\lambda_{\theta }^{2}}{\cos^{2}\varphi }\right)}, \end{array}$$

being one of the equations of motion derived from PMP in the energy-optimal case (see Section 3).

From the fact that $\varphi \left(T/2\right)\equiv \varphi_{0}$ is maximum at t=T/2, we plug the identity $\dot{\varphi }\left(t=T/2\right)=0$ into (26), which gives the relation:

(A7) $$\alpha \sin^{4}\varphi_{0}-\beta \sin^{2}\varphi_{0}+1=0,$$

with the parameters:

(A8) $$\alpha =\frac{\mu }{h},\;\beta =\frac{2h+2\mu +\lambda_{\theta }^{2}}{2h},$$

as a function of which one can express two possible values of $\sin^{2}\varphi_{0}$:

(A9) $$\begin{array}{c} \sin^{2}\varphi_{0,\pm }=\frac{1}{2\alpha }\left(\beta \pm \sqrt{\beta^{2}-4\alpha }\right). \end{array}$$

In general, this solution exists if $\beta^{2}\geq 4\alpha$ and $(\beta \pm \sqrt{\beta^{2}-4\alpha })/\alpha >0$. If α>0 and $\beta^{2}>4\alpha$, there are two roots $\varphi_{0,\pm }$. If α<0 and β<0, only $\varphi_{0,+}$ exists. If α<0 and β>0, only $\varphi_{0,-}$ exists.

The differential Equation (26) can be rewritten as:

(A10) $$\begin{array}{cc} \dot{\varphi }\left|\cos\varphi \right| & =\pm \sqrt{2h-\left(2h+2\mu +\lambda_{\theta }^{2}\right)\sin^{2}\varphi +2\mu \sin^{4}\varphi }. \end{array}$$

which gives, for 0≤φ≤π/2:

(A11) $$\begin{array}{cc} \frac{d\sin\varphi }{\sqrt{1-\beta \sin^{2}\varphi +\alpha \sin^{4}\varphi }} & =\pm \sqrt{2h}\;dt, \end{array}$$

taking h≥0. Integrating the positive branch (0≤t≤T/2, $0\leq \sin\varphi \leq \sin\varphi_{0}$) leads to:

(A12) $$\begin{array}{cc} \int_{0}^{\sin\varphi }\frac{ds}{\sqrt{1-\beta s^{2}+\alpha s^{4}}} & =\sqrt{2h}\;t. \end{array}$$

When μ=0 (i.e., α=0), the integration leads for the positive branch (0≤t≤T/2):

(A13) $$\begin{array}{c} \sin\varphi =\sin\varphi_{0}\sin\left(\frac{\sqrt{2h}}{\sin\varphi_{0}}t\right)=\sin\varphi_{0}\sin\left(\frac{\lambda_{\theta }}{\cos\varphi_{0}}t\right), \end{array}$$

implying at t=T/2:

(A14) $$\lambda_{\theta }T=\pi \cos\varphi_{0},$$

and giving:

(A15) $$\begin{array}{c} \sin\varphi =\sin\varphi_{0}\sin\left(\pi t/T\right). \end{array}$$

By imposing the symmetry in Equation (27), $\theta (\varphi_{0})=\pi /4$, we obtain $\varphi_{0}=\pi /3$ for μ=0.

When μ≠0, the differential Equation (A12) involves an incomplete elliptic integral of the first kind:

(A16) $$F\left(\Phi |m\right)=\int_{0}^{\Phi }\frac{d\vartheta }{\sqrt{1-m\sin^{2}\vartheta }},$$

defined for −π/2<Φ<π/2, and $m\sin^{2}\Phi <1$, 0≤ϑ≤Φ, as follows: defining s=κsinϑ, giving ds=dϑκcosϑ with cosϑ≥0, i.e., $d\vartheta =ds/(\kappa \sqrt{1-{\left(s/\kappa \right)}^{2}})$, we obtain:

(A17) $$\begin{array}{cc} \kappa F(\Phi |m)\; & =\int_{0}^{\kappa \sin\Phi }\frac{ds}{\sqrt{1-\left(m+1\right)s^{2}/\kappa^{2}+ms^{4}/\kappa^{4}}}. \end{array}$$

By identification with (A12), we have:

(A18) $$\beta =\left(m+1\right)/\kappa^{2},\;\alpha =m/\kappa^{4},$$

and sinφ=κsinΦ. We impose the symmetry $\varphi =\varphi_{0}$ for Φ=π/2 (for which the elliptic integral of the first kind is complete), i.e.,

(A19) $$\begin{array}{cc} \kappa & =\sin\varphi_{0}, \end{array}$$

and, for the positive branch:

(A20) $$\begin{array}{c} F\left(\sin^{-1}\left(\sin\varphi /\sin\varphi_{0}\right)|m\right)\sin\varphi_{0}=\sqrt{2h}\;t. \end{array}$$

with, at time t=T/2:

(A21) $$\begin{array}{cc} \; & F\left(\pi /2|m\right)\equiv K\left(m\right)=\frac{\sqrt{2h}\;T}{2\sin\varphi_{0}}, \end{array}$$

where K(m) is the complete elliptic integral of the first kind. This gives a condition on $\sqrt{2h}/\sin\varphi_{0}$, which we insert into (A20):

(A22) $$\begin{array}{c} F\left(\sin^{-1}\left(\sin\varphi /\sin\varphi_{0}\right)|m\right)=2K\left(m\right)\;t/T. \end{array}$$

The function K(m) has the property to be real (and positive) when m<1, and K(m)→∞ when m→1. The Jacobi elliptic functions allow one to inverse the incomplete elliptic integral of the first kind with respect to their first argument:

(A23) $$\begin{array}{c} \mathrm{sn}(F(\Phi |m),m)=\sin\Phi ,\;\mathrm{cn}(F(\Phi |m),m)=\cos\Phi , \end{array}$$

and (A22) becomes (to be compared to (A15) when μ=0 using F(Φ|0)=Φ):

(A24) $$\begin{array}{c} \sin\varphi =\sin\varphi_{0}\;\mathrm{sn}\left(2K\left(m\right)\;t/T,m\right), \end{array}$$

where the property sn(K(m),m)=1 ensures that $\varphi \left(t=T/2\right)=\varphi_{0}$.

This shows that the set of the three parameters $\left\{\mu ,\lambda_{\theta },h\right\}$, or {α,β,h} from (A8), can be replaced by the set $\left\{m,\varphi_{0},T\right\}$. The correspondence between the last two sets is as follows: from a given α and β, the parameters κ (and thus, $\varphi_{0}$ from Equation (A19)) and *m* are determined from Equation (A18). Equation (A21) gives *T* from the additional knowledge of *h*.

## Appendix C. Integration of Equation (27)

In this Appendix, we solve the differential Equation (27):

(A25) $$\begin{array}{cc} \frac{d\varphi }{d\theta } & =\pm \sqrt{2h}\frac{\sqrt{1-\beta \sin^{2}\varphi +\alpha \sin^{4}\varphi }}{\lambda_{\theta }\cos\varphi \tan^{2}\varphi }, \end{array}$$

being the second equation of motion derived from PMP in the energy-optimal case (see Section 3). The sign ± is the same as the one of $\dot{\varphi }$, which, taking into account that θ=0 when φ=0, leads to:

(A26) $$\begin{array}{cc} \theta & =\pm \frac{\sqrt{\sin^{2}\varphi_{0}-m}}{\sin\varphi_{0}\tan\varphi_{0}}\int_{0}^{\varphi }\frac{d\phi }{\sqrt{1-\beta \sin^{2}\phi +\alpha \sin^{4}\phi }}\frac{\sin^{2}\phi }{\cos\phi }. \end{array}$$

From the result:

(A27) $$\begin{array}{cc} \; & \int_{0}^{\varphi }d\phi \frac{\sin^{2}\phi }{\cos\phi \sqrt{\left(1-a\sin^{2}\phi \right)\left(1-b\sin^{2}\phi \right)}}=\frac{1}{\sqrt{a-b}}[F\left(\mathrm{asin}\left(\sqrt{1-a\sin^{2}\varphi }\right)\frac{b}{b-a}\right)\\ \; & -K\left(\frac{b}{b-a}\right)+\frac{a}{a-1}\Pi \left(\frac{1}{1-a};-\mathrm{asin}\left(\sqrt{1-a\sin^{2}\varphi }\right)\frac{b}{b-a}\right)+\frac{a}{a-1}\Pi \left(\frac{1}{1-a}\frac{b}{b-a}\right)], \end{array}$$

where Π(n;Φ|m) is an incomplete elliptic integral of the third kind:

(A28) $$\Pi \left(n;\Phi |m\right)=\int_{0}^{\Phi }\frac{d\phi }{\left(1-n\sin^{2}\phi \right)\sqrt{1-m\sin^{2}\phi }},$$

we identify:

(A29) $$\begin{array}{c} \beta =a+b,\;\alpha =ab, \end{array}$$

which gives:

(A30) $$\begin{array}{cc} b & =\frac{1}{2}\left(\beta \pm \sqrt{\beta^{2}-4\alpha }\right)=\alpha \sin^{2}\varphi_{0,\pm }, \end{array}$$

and Equation (A26) becomes for the positive branch (for which φ increases from 0 to $\varphi_{0}$):

(A31) $$\begin{array}{cc} \; & \theta_{+}\left(\varphi \right)=\frac{\sqrt{\sin^{2}\varphi_{0}-m}}{\sqrt{1-m}\;\tan\varphi_{0}}[F\left(\mathrm{asin}\sqrt{1-\frac{\sin^{2}\varphi }{\sin^{2}\varphi_{0}}}\frac{m}{m-1}\right)-K\left(\frac{m}{m-1}\right)\\ \; & +\frac{\Pi \left(-\tan^{2}\varphi_{0};-\mathrm{asin}\sqrt{1-\frac{\sin^{2}\varphi }{\sin^{2}\varphi_{0}}}\frac{m}{m-1}\right)}{\cos^{2}\varphi_{0}}+\frac{\Pi \left(-\tan^{2}\varphi_{0}\frac{m}{m-1}\right)}{\cos^{2}\varphi_{0}}]. \end{array}$$

Imposing the symmetrical negative branch:

(A32) $$\theta_{-}\left(\varphi \right)=\frac{\pi }{2}-\theta_{+}\left(\varphi \right),\;\theta_{-}\left(\varphi_{0}\right)=\theta_{+}\left(\varphi_{0}\right)=\frac{\pi }{4},$$

as in the unconstrained case μ=0, leads to:

(A33) $$\begin{array}{cc} \; & \frac{\pi }{4}=+\frac{\sqrt{\sin^{2}\varphi_{0}-m}}{\sqrt{1-m}\;\tan\varphi_{0}}\left[-K\left(\frac{m}{m-1}\right)+\frac{\Pi \left(-\tan^{2}\varphi_{0}\frac{m}{m-1}\right)}{\cos^{2}\varphi_{0}}\right], \end{array}$$

which gives an implicit relation between $\sin\varphi_{0}$ and *m*.

We can recover for μ=0 (i.e., α=0 and m=0) $\varphi_{0}=\pi /3$ (solution of (33)), giving $\tan\varphi_{0}\equiv \sqrt{3}=\frac{\sqrt{2h}}{\lambda_{\theta }}$, i.e., a=0 and b=4/3, and the positive branch:

(A34) $$\theta_{+}=\mathrm{atan}\left(\frac{\sin\varphi_{+}}{\sqrt{\tan^{2}\varphi_{0}-\frac{\sin^{2}\varphi_{+}}{\cos^{2}\varphi_{0}}}}\right)-\cos\varphi_{0}\mathrm{asin}\left(\frac{\sin\varphi_{+}}{\sin\varphi_{0}}\right).$$

Using:

(A35) $$\mathrm{atan}\left(x\right)=\mathrm{asin}\left(\frac{\tan\varphi_{+}}{\tan\varphi_{0}}\right),$$

we obtain for μ=0:

(A36) $$\theta_{+}=\mathrm{asin}\left(\frac{\tan\varphi_{+}}{\tan\varphi_{0}}\right)-\cos\varphi_{0}\mathrm{asin}\left(\frac{\sin\varphi_{+}}{\sin\varphi_{0}}\right).$$

## Appendix D. Stimulated Raman Parallel Adiabatic Passage

In this Appendix, we briefly summarize the stimulated Raman parallel adiabatic passage [28] with coincident pulses. For such a process, we have to consider quasi-resonant pulses:

(A37) $$H=\left[\begin{array}{ccc} -\delta /2 & u_{P} & 0\\ u_{P} & \Delta & u_{S}\\ 0 & u_{S} & \delta /2 \end{array}\right],\;$$

with $\;u_{p}\equiv \Omega_{P}/2$, $u_{s}\equiv \Omega_{S}/2$, and δ the two-photon detuning: $\delta =\omega_{3}-\omega_{1}-\omega_{P}+\omega_{S}$, Δ connected to the one-photon detuning (with respect to the pump) $\Delta_{P}=\omega_{2}-\omega_{1}-\omega_{P}$ as $\Delta =\Delta_{P}-\delta /2$. $\hbar \omega_{j}$, j=1,2,3, are the energies of the corresponding state |j〉 and $\omega_{P}$, $\omega_{S}$ the frequencies of the pump and Stokes fields, respectively. Parallel STIRAP [28] requires an adiabatic passage process such that the eigenvalues stay parallel at each time, implying minimization of non-adiabatic transfers (still in the adiabatic limit) [29]. One denotes $\omega_{-,0,+}$ the three eigenvalues which satisfy $\omega_{-}<\omega_{0}<\omega_{+}$ and $|\psi_{-,0,+}\rangle$, with corresponding eigenstates such that $\left|1\rangle \overset{t\to -\infty }{\;⟵}\right|\psi_{0}\rangle \overset{t\to +\infty }{\;⟶}|3\rangle$. The eigenvalues, on which we impose parallelism: $\omega_{+}-\omega_{0}=\omega_{0}-\omega_{-}=\Omega_{0}/2$, are, thus, of the form:

(A38) $$\omega_{0}=\frac{1}{3}\Delta ,\;\omega_{\pm }=\frac{1}{3}\Delta \pm \frac{1}{2}\Omega_{0},$$

with

(A39) $$\Omega_{0}=\sqrt{\Omega_{P}^{2}+\Omega_{S}^{2}+\delta^{2}+\frac{4}{3}\Delta^{2}}$$

and the condition

(A40) $$0=\frac{\delta }{2}\left(\Omega_{S}^{2}-\Omega_{P}^{2}\right)+\frac{\Delta }{3}\left(\Omega_{P}^{2}+\Omega_{S}^{2}\right)+\frac{\Delta }{27}\left(8\Delta^{2}-18\delta^{2}\right).$$

We choose the initial and final connections, when $\Omega_{P}=\Omega_{S}=0$, $\omega_{+}\left(-\infty \right)=\Omega_{0}/4$, $\omega_{-}\left(-\infty \right)=-3\Omega_{0}/4$, $\omega_{+}\left(+\infty \right)=3\Omega_{0}/4$, $\omega_{-}\left(+\infty \right)=-\Omega_{0}/4$ corresponding to the final and initial conditions: $0\overset{t\to -\infty }{\;⟵}\Omega_{P,S}\left(t\right)\overset{t\to +\infty }{\;⟶}0$, $-3\Omega_{0}/4\overset{t\to -\infty }{\;⟵}\Delta \left(t\right)\overset{t\to +\infty }{\;⟶}3\Omega_{0}/4$, $\Omega_{0}/2\overset{t\to -\infty }{\;⟵}\delta \left(t\right)\overset{t\to +\infty }{\;⟶}\Omega_{0}/2$.

Considering coincident pulses: $\Omega_{P}\left(t\right)=\Omega_{S}\left(t\right)\equiv \Omega \left(t\right)$ (i.e., $u_{P}\left(t\right)=u_{S}\left(t\right)\equiv \Omega \left(t\right)/2$), we obtain expressions for the two-photon detuning and the pulses from (A39) and (A40):

(A41) $$\begin{array}{c} \delta =\frac{\Omega_{0}}{\sqrt{3}}\sqrt{1-{\left(\frac{2}{3}\frac{\Delta }{\Omega_{0}}\right)}^{2}},\;\Omega =\frac{\Omega_{0}}{\sqrt{3}}\sqrt{1-{\left(\frac{4}{3}\frac{\Delta }{\Omega_{0}}\right)}^{2}}, \end{array}$$

considering δ(t) and Ω(t) as functions of Δ(t). Taking for simplicity a monotonic increasing odd function $\Delta \left(t\right)=\left(3\Omega_{0}/4\right)g\left(t\right)$, with g(±∞)=±1, g(0)=0, for instance, g(t)=erf(t/T) i.e.,

(A42) $$\Delta \left(t\right)=\frac{3\Omega_{0}}{4}\mathrm{erf}\left(\frac{t}{T}\right)$$

with *T* the characteristic time of evolution, we obtain:

(A43) $$\begin{array}{c} \delta =\frac{\Omega_{0}}{\sqrt{3}}\sqrt{1-\frac{1}{4}{\left[\mathrm{erf}\left(\frac{t}{T}\right)\right]}^{2}},\;\Omega =\frac{\Omega_{0}}{\sqrt{3}}\sqrt{1-{\left[\mathrm{erf}\left(\frac{t}{T}\right)\right]}^{2}}, \end{array}$$

i.e.,

(A44) $$\begin{array}{c} u_{P}=u_{S}\equiv \frac{\Omega }{2}=\frac{\Omega_{0}}{2\sqrt{3}}\sqrt{1-{\left[\mathrm{erf}\left(\frac{t}{T}\right)\right]}^{2}}. \end{array}$$

## Appendix E. Optimal Control with Constrained Controls

We consider a control pseudo-Hamiltonian of the bilinear form [32]:

(A45) $$H_{c}=H_{0}+u_{1}H_{1}+u_{2}H_{2},$$

where $H_{0}$, $H_{1}$, and $H_{2}$ are independent of the controls $u_{1}$, $u_{2}$, and a constraint on the controls:

(A46) $$u_{1}^{2}+u_{2}^{2}\leq u_{0}^{2}.$$

The goal is to maximize $H_{c}$ for any value of $H_{0}$, $H_{1}$, $H_{2}$, under the above constraint. The maximization of $H_{c}$ corresponds to the necessary conditions $\frac{\partial H_{c}}{\partial u_{1}}=0$, $\frac{\partial H_{c}}{\partial u_{2}}=0$ according to the PMP, in the case of absence of constraint on the controls, but this is no longer true with a constraint.

Keeping the full generality of the problem, we can rewrite the controls as follows:

(A47) $$\begin{array}{c} u_{1}\left(t\right)=u_{m}\left(t\right)\cos\left(\theta (t)\right),\;u_{2}\left(t\right)=u_{m}\left(t\right)\sin\left(\theta (t)\right), \end{array}$$

implying

(A48) $$u_{1}^{2}\left(t\right)+u_{2}^{2}\left(t\right)=u_{m}^{2}\left(t\right)\leq u_{0}^{2}.$$

This means that the constraint condition (A46) is transferred to the condition (A48) on $u_{m}$: $u_{m}^{2}\leq u_{0}^{2}$, which is independent of θ. The maximization of $H_{c}$ corresponds, thus, to the necessary condition:

(A49) $$\frac{\partial H_{c}}{\partial \theta }=0,$$

which gives:

(A50) $$\frac{\partial H_{c}}{\partial \theta }=\frac{\partial u_{1}}{\partial \theta }H_{1}+\frac{\partial u_{2}}{\partial \theta }H_{2}=u_{m}\left(H_{2}\cos\theta -H_{1}\sin\theta \right)=0,$$

i.e.,

(A51) $$\frac{H_{2}}{H_{1}}=\frac{\sin\theta }{\cos\theta },$$

or

(A52) $$\begin{array}{c} \cos\theta =\frac{H_{1}}{\sqrt[{}]{H_{1}^{2}+H_{2}^{2}}},\;\sin\theta =\frac{H_{2}}{\sqrt[{}]{H_{1}^{2}+H_{2}^{2}}}, \end{array}$$

and yield

(A53) $$\begin{array}{c} u_{1}=u_{m}\frac{H_{1}}{\sqrt[{}]{H_{1}^{2}+H_{2}^{2}}},\;u_{2}=u_{m}\frac{H_{2}}{\sqrt[{}]{H_{1}^{2}+H_{2}^{2}}}. \end{array}$$

We plug them into the pseudo-Hamiltonian equation:

(A54) $$\begin{array}{cc} H_{c} & =H_{0}+u_{m}\;\sqrt[{}]{H_{1}^{2}+H_{2}^{2}}. \end{array}$$

The pseudo-Hamiltonian equation is then maximum when $u_{m}$ takes its maximum value, i.e., according to (A46):

(A55) $$u_{m}=u_{0},$$

and it finally reads:

(A56) $$H_{c}=H_{0}+u_{0}\;\sqrt[{}]{H_{1}^{2}+H_{2}^{2}}$$

with the controls:

(A57) $$\begin{array}{c} u_{1}=u_{0}\frac{H_{1}}{\sqrt[{}]{H_{1}^{2}+H_{2}^{2}}},\;u_{2}=u_{0}\frac{H_{2}}{\sqrt[{}]{H_{1}^{2}+H_{2}^{2}}}. \end{array}$$

One can remark that the controls attain the maximum of the constraint at each time:

(A58) $$u_{1}^{2}\left(t\right)+u_{2}^{2}\left(t\right)=u_{0}^{2}.$$

## Appendix F. Roots of Equation (91)

When $\tilde{\mu }\neq 0$, and multiplying Equation (91) by $1-\sin^{2}\varphi_{0}$, we obtain that $\sin^{2}\varphi_{0}$ is a (positive and less than (or equal to) one) root of a cubic polynomias:

(A59) $$1-\left(1+{\tilde{\lambda }}_{\theta }^{2}+2\tilde{\mu }\right)\sin^{2}\varphi_{0}+\left({\tilde{\mu }}^{2}+2\tilde{\mu }\right)\sin^{4}\varphi_{0}-{\tilde{\mu }}^{2}\sin^{6}\varphi_{0}=0,$$

i.e.,

(A60) $$1-\left(1+{\tilde{\lambda }}_{\theta }^{2}+2\tilde{\mu }\right)X+\left({\tilde{\mu }}^{2}+2\tilde{\mu }\right)X^{2}-{\tilde{\mu }}^{2}X^{3}=0$$

with $X=\sin^{2}\varphi_{0}$. It is important to note that multiplying Equation (91) by $1-\sin^{2}\varphi_{0}$ introduces an artificial root $\varphi_{0}=\pi /2$, except for ${\tilde{\lambda }}_{\theta }=0$ and $\tilde{\mu }=1$, for which $\varphi_{0}=\pi /2$ is a true root of (91). We analyze the solution $\varphi_{0}$ near π/2 by setting $\varphi_{0}=\pi /2+\varepsilon$, |ε|≪1, into (91), which gives a relation between ${\tilde{\lambda }}_{\theta }$ and $\tilde{\mu }$ for a given ε:

(A61) $$|{\tilde{\lambda }}_{\theta }|=|\varepsilon |\times |1-\tilde{\mu }|,$$

which shows how ${\tilde{\lambda }}_{\theta }$ goes to zero and $\varphi_{0}$ to π/2 for a given $\tilde{\mu }$.

When $\tilde{\mu }=0$, we obtain:

(A62) $$\varphi_{0}=\arctan\frac{1}{{\tilde{\lambda }}_{\theta }}.$$

One can use the analytic Cardano’s method to solve Equation (A60), as shown below. Substituting the following:

(A63) $$\begin{array}{c} X=Y+\frac{{\tilde{\mu }}^{2}+2\tilde{\mu }}{3{\tilde{\mu }}^{2}}=Y+\frac{\tilde{\mu }+2}{3\tilde{\mu }}, \end{array}$$

Equation (A60) can be depressed to:

(A64) $$Y^{3}+pY+q=0,$$

with $p=\frac{3{\tilde{\lambda }}_{\theta }^{2}-1+2\tilde{\mu }-{\tilde{\mu }}^{2}}{3{\tilde{\mu }}^{2}}$, $q=\frac{2+9\left(\tilde{\mu }+2\right){\tilde{\lambda }}_{\theta }^{2}-6\tilde{\mu }+6{\tilde{\mu }}^{2}-2{\tilde{\mu }}^{3}}{27{\tilde{\mu }}^{3}}$. We define the discriminant of Equation (A64) as follows:

(A65) $$\Delta ={\left(\frac{q}{2}\right)}^{2}+{\left(\frac{p}{3}\right)}^{3}.$$

When Δ>0, the only real solution of Equation (A64) reads:

(A66) $$\begin{array}{c} Y=\sqrt[{3}]{\sqrt{{\left(\frac{q}{2}\right)}^{2}+{\left(\frac{p}{3}\right)}^{3}}-\frac{q}{2}}+\sqrt[{3}]{-\sqrt{{\left(\frac{q}{2}\right)}^{2}+{\left(\frac{p}{3}\right)}^{3}}-\frac{q}{2}}, \end{array}$$

where the cube root is used (imposing a real argument).

When Δ≤0, which implies that *p* is negative, the solutions of Equation (A64) read (being all real):

(A67) $$Y_{k+1}=2\sqrt{\frac{-p}{3}}\cos\left(\frac{1}{3}\arccos\left(\frac{3q}{2p}\sqrt{\frac{3}{-p}}\right)+\frac{2k\pi }{3}\right)$$

with k∈{0,1,2}. The relationship between the roots and the coefficients is as follows:

(A68) $$\begin{array}{cc} Y_{1}+Y_{2}+Y_{3} & =0, \end{array}$$

(A69) $$\begin{array}{cc} \frac{1}{Y_{1}}+\frac{1}{Y_{2}}+\frac{1}{Y_{3}} & =-\frac{p}{q}, \end{array}$$

(A70) $$\begin{array}{cc} Y_{1}Y_{2}Y_{3} & =-q. \end{array}$$

When Δ=0 (implying p≤0), Equation (A64) has two roots when p,q≠0:

(A71) $$\begin{array}{c} Y_{1}=2\sqrt[{3}]{-\frac{q}{2}},\;Y_{2}=\sqrt[{3}]{\frac{q}{2}}=Y_{3}, \end{array}$$

and three being equal to zero when q=0 (implying p=0).

We are searching for positive and real roots, satisfying |Y|≤1. We first determine the sign of the discriminant:

(A72) $$\begin{array}{cc} \Delta = & \frac{{\tilde{\lambda }}_{\theta }^{2}}{27{\tilde{\mu }}^{6}}\left[{\tilde{\lambda }}_{\theta }^{4}+\left(-{\tilde{\mu }}^{2}/4+5\tilde{\mu }+2\right){\tilde{\lambda }}_{\theta }^{2}+1-3\tilde{\mu }+3{\tilde{\mu }}^{2}-{\tilde{\mu }}^{3}\right]\equiv \frac{{\tilde{\lambda }}_{\theta }^{2}}{27{\tilde{\mu }}^{6}}P_{2}\left({\tilde{\lambda }}_{\theta }^{2}\right), \end{array}$$

where we denote the two (positive) roots of $P_{2}\left({\tilde{\lambda }}_{\theta }^{2}\right)$:

(A73) $$\begin{array}{cc} {\tilde{\lambda }}_{\theta ,\pm }^{2} & =\frac{1}{2}\left(\frac{1}{4}{\tilde{\mu }}^{2}-5\tilde{\mu }-2\pm \frac{1}{4}\sqrt{\tilde{\mu }{\left(\tilde{\mu }+8\right)}^{3}}\right). \end{array}$$

The sign of Δ is given by the sign of the second-order polynomial $P_{2}\left({\tilde{\lambda }}_{\theta }^{2}\right)$ in ${\tilde{\lambda }}_{\theta }^{2}$. We first note that Δ=0 when ${\tilde{\lambda }}_{\theta }=0$.

When Δ>0, the real solution reads:

(A74) $$\begin{array}{cc} \sin^{2}\varphi_{0} & =\sqrt[{3}]{-\frac{q}{2}+\frac{{\tilde{\lambda }}_{\theta }}{\sqrt{27}{\tilde{\mu }}^{3}}\sqrt{P_{2}\left({\tilde{\lambda }}_{\theta }^{2}\right)}}+\sqrt[{3}]{-\frac{q}{2}-\frac{{\tilde{\lambda }}_{\theta }}{\sqrt{27}{\tilde{\mu }}^{3}}\sqrt{P_{2}\left({\tilde{\lambda }}_{\theta }^{2}\right)}}+\frac{\tilde{\mu }+2}{3\tilde{\mu }}. \end{array}$$

When Δ=0, the two solutions read:

(A75) $$\begin{array}{cc} \sin^{2}\varphi_{0} & =2\sqrt[{3}]{-\frac{q}{2}}+\frac{\tilde{\mu }+2}{3\tilde{\mu }}, \end{array}$$

(A76) $$\begin{array}{cc} \sin^{2}\varphi_{0} & =-\sqrt[{3}]{-\frac{q}{2}}+\frac{\tilde{\mu }+2}{3\tilde{\mu }}=\sqrt[{3}]{\frac{q}{2}}+\frac{\tilde{\mu }+2}{3\tilde{\mu }}. \end{array}$$

When Δ<0, the three real solutions read (with k∈{0,1,2}):

(A77) $$\begin{array}{cc} \sin^{2}\varphi_{0}= & 2\sqrt{\frac{-p}{3}}\cos\left(\frac{1}{3}\arccos\left(\frac{3q}{2p}\sqrt{\frac{3}{-p}}\right)+\frac{2k\pi }{3}\right)+\frac{\tilde{\mu }+2}{3\tilde{\mu }}. \end{array}$$

We have, thus, the following real roots:

• When ${\tilde{\lambda }}_{\theta }=0$, according to (91),
  -one root, when $\tilde{\mu }\geq 1$:
  (A78) $$\begin{array}{cc} \sin^{2}\varphi_{0} & =\frac{1}{\tilde{\mu }}, \end{array}$$
  -no root, when $\tilde{\mu }<1$;
• When ${\tilde{\lambda }}_{\theta }\neq 0$:
  -for $\tilde{\mu }\leq -8$:
  one root (A74), for ${\tilde{\lambda }}_{\theta }\in ]0,{\tilde{\lambda }}_{\theta ,-}\left[\cup \right]{\tilde{\lambda }}_{\theta ,+},+\infty [$,
  two roots (A75) and (A76), for ${\tilde{\lambda }}_{\theta }={\tilde{\lambda }}_{\theta ,-}$ or ${\tilde{\lambda }}_{\theta }={\tilde{\lambda }}_{\theta ,+}$,
  three roots (A77), for ${\tilde{\lambda }}_{\theta ,-}<{\tilde{\lambda }}_{\theta }<{\tilde{\lambda }}_{\theta ,+}$;
  -for $\tilde{\mu }\in ]-8,1]$: one root (A74) (which coincides with (A62) for $\tilde{\mu }=0$);
  -for $\tilde{\mu }>1$:
  one root (A74), for ${\tilde{\lambda }}_{\theta }>{\tilde{\lambda }}_{\theta ,+}$,
  two roots (A75) and (A76), for ${\tilde{\lambda }}_{\theta }={\tilde{\lambda }}_{\theta ,+}$,
  three roots (A77), for $0<{\tilde{\lambda }}_{\theta }<{\tilde{\lambda }}_{\theta ,+}$.

One can analyze the limit when ${\tilde{\lambda }}_{\theta }$ goes to zero:

- In the range $\tilde{\mu }\leq 1$, the root given by (A74), where, for ${\tilde{\lambda }}_{\theta }=0$:
  (A79) $$\begin{array}{c} q=\frac{2}{27{\tilde{\mu }}^{3}}{\left(1-\tilde{\mu }\right)}^{3},\;p=-\frac{1}{3}\frac{{\left(1-\tilde{\mu }\right)}^{2}}{{\tilde{\mu }}^{2}} \end{array}$$
  gives:
  (A80) $$\sin^{2}\varphi_{0}=1,$$
  which is compatible with $\varphi_{0}$ going to π/2 for ${\tilde{\lambda }}_{\theta }$ going to 0.
- In the range $\tilde{\mu }>1$, Equation (A77):
  (A81) $$\begin{array}{cc} \sin^{2}\varphi_{0}\; & =\frac{2}{3}\frac{\tilde{\mu }-1}{\tilde{\mu }}\cos\left(\frac{2k\pi }{3}\right)+\frac{\tilde{\mu }+2}{3\tilde{\mu }} \end{array}$$
  gives the two roots:
  (A82) $$\sin^{2}\varphi_{0}=\left\{1,1/\tilde{\mu }\right\}.$$

Contour plots as a function of $\tilde{\mu }$ and ${\tilde{\lambda }}_{\theta }$ in Figure A1 show the solution(s) of $\varphi_{0}$ satisfying (91). Considering that the maximum number of solutions is three, we make three plots.

## Appendix G. Lindblad Equation

In this Appendix, we consider explicitly all the decay channels via the Lindblad Equation [33]:

(A83) $$\frac{d\rho }{dt}=-i\left[H\left(t\right),\rho \left(t\right)\right]+D\left(\rho \left(t\right)\right)$$

with the dissipator:

(A84) $$D\left(\rho \left(t\right)\right)=\sum_{i}\left[\gamma_{i}\sigma_{i}\rho \left(t\right)\sigma_{i}^{†}-\frac{\gamma_{i}}{2}\left(\sigma_{i}^{†}\sigma_{i}\rho \left(t\right)+\rho \left(t\right)\sigma_{i}^{†}\sigma_{i}\right)\right],$$

where $\sigma_{i}$ is the jump operator associated with the rate $\gamma_{i}$ and $\rho^{†}\left(t\right)=\rho \left(t\right)$ is the density operator [34]. The populations $\rho_{jj}=\langle j|\rho |j\rangle$ satisfy $\sum_{j}\rho_{jj}=1$ and the coherences $\rho_{ij}^{*}=\rho_{ji}$. In the basis {|1〉,|3〉,|e〉,|a〉}, the (non-lossy) resonantly driven Hamiltonian reads:

(A85) $$H=\left[\begin{array}{cccc} 0 & 0 & u_{p} & 0\\ 0 & 0 & u_{s} & 0\\ u_{p} & u_{s} & 0 & 0\\ 0 & 0 & 0 & 0 \end{array}\right].$$

The Λ system features in general (i) two decay paths within the Λ-system, $\gamma_{1}$ and $\gamma_{3}$, between the upper state denoted |e〉≡|2〉 and the two ground states |1〉 and |3〉, respectively, with the corresponding jump operator:

(A86) $$\sigma_{je}=|j\rangle \langle e|,$$

and (ii) additional decays from state |e〉 outside the Λ system, which can be modeled by an imaginary loss of the form −iΓ/2. In order to show it, one considers an additional state |a〉 gathering the additional decays $\gamma_{a}$ with the corresponding jump operator $\sigma_{ae}=\left|a\rangle \langle e\right|$, and we decompose the dissipator into the respective terms:

(A87) $$\begin{array}{cc} D(\rho (t)) & =D_{13}\left(\rho \left(t\right)\right)+D_{a}\left(\rho \left(t\right)\right) \end{array}$$

with:

(A88) $$\begin{array}{cc} D_{13}\left(\rho \left(t\right)\right) & =\sum_{j=1,3}\left[\gamma_{j}\sigma_{je}\rho \left(t\right)\sigma_{je}^{†}-\frac{\gamma_{j}}{2}\left(\sigma_{je}^{†}\sigma_{je}\rho \left(t\right)+\rho \left(t\right)\sigma_{je}^{†}\sigma_{je}\right)\right], \end{array}$$

(A89) $$\begin{array}{cc} D_{a}\left(\rho \left(t\right)\right) & =\gamma_{a}\sigma_{ae}\rho \left(t\right)\sigma_{ae}^{†}-\frac{\gamma_{a}}{2}\left(\sigma_{ae}^{†}\sigma_{ae}\rho \left(t\right)+\rho \left(t\right)\sigma_{ae}^{†}\sigma_{ae}\right) \end{array}$$

with:

(A90) $$\begin{array}{cc} \; & \sigma_{je}\rho \sigma_{je}^{†}=\rho_{ee}|j\rangle \langle j|, \end{array}$$

(A91) $$\begin{array}{cc} \; & \sigma_{je}^{†}\sigma_{je}\rho =|e\rangle \langle e|\rho =\left|e\rangle \langle e\right|\sum_{k\ell }\rho_{k\ell }\left|k\rangle \langle \ell \right|=\sum_{\ell }\rho_{e\ell }|e\rangle \langle \ell |, \end{array}$$

(A92) $$\begin{array}{cc} \; & \rho \sigma_{je}^{†}\sigma_{je}=\rho |e\rangle \langle e|=\sum_{k}\rho_{ke}|k\rangle \langle e|, \end{array}$$

i.e., in matrix form:

(A93) $$\begin{array}{cc} D_{13}\left(\rho \left(t\right)\right) & =\left[\begin{array}{cccc} \gamma_{1}\rho_{ee} & 0 & -\frac{\gamma_{1}+\gamma_{3}}{2}\rho_{1e} & 0\\ 0 & \gamma_{3}\rho_{ee} & -\frac{\gamma_{1}+\gamma_{3}}{2}\rho_{3e} & 0\\ -\frac{\gamma_{1}+\gamma_{3}}{2}\rho_{e1} & -\frac{\gamma_{1}+\gamma_{3}}{2}\rho_{e3} & -\left(\gamma_{1}+\gamma_{3}\right)\rho_{ee} & -\frac{\gamma_{1}+\gamma_{3}}{2}\rho_{ea}\\ 0 & 0 & -\frac{\gamma_{1}+\gamma_{3}}{2}\rho_{ae} & 0 \end{array}\right], \end{array}$$

(A94) $$\begin{array}{cc} D_{a}\left(\rho \left(t\right)\right) & =\left[\begin{array}{cccc} 0 & 0 & -\frac{\gamma_{a}}{2}\rho_{1e} & 0\\ 0 & 0 & -\frac{\gamma_{a}}{2}\rho_{3e} & 0\\ -\frac{\gamma_{a}}{2}\rho_{e1} & -\frac{\gamma_{a}}{2}\rho_{e3} & -\gamma_{a}\rho_{ee} & -\frac{\gamma_{a}}{2}\rho_{ea}\\ 0 & 0 & -\frac{\gamma_{a}}{2}\rho_{ae} & \gamma_{a}\rho_{ee} \end{array}\right]. \end{array}$$

We introduce the lossy Hamiltonian equation:

(A95) $$H_{\gamma_{a}}=H-i\frac{\gamma_{a}}{2}\sigma_{ae}^{†}\sigma_{ae},$$

i.e., in matrix form:

(A96) $$H_{\gamma_{a}}=\left[\begin{array}{cccc} 0 & 0 & u_{p} & 0\\ 0 & 0 & u_{s} & 0\\ u_{p} & u_{s} & -i\frac{\gamma_{a}}{2} & 0\\ 0 & 0 & 0 & 0 \end{array}\right],$$

satisfying:

(A97) $$H_{\gamma_{a}}^{†}=H+i\frac{\gamma_{a}}{2}\sigma_{ae}^{†}\sigma_{ae}=H_{-\gamma_{a}}.$$

Using the above property in the second term of the commutator, we can expand without any approximation the Lindblad equation using the lossy Hamiltonian equation as follows:

(A98) $$\begin{array}{cc} \frac{d\rho }{dt} & =-i\left(H_{\gamma_{a}}+i\frac{\gamma_{a}}{2}\sigma_{ae}^{†}\sigma_{ae}\right)\rho \left(t\right)+i\rho \left(t\right)\left(H_{\gamma_{a}}^{†}-i\frac{\gamma_{a}}{2}\sigma_{ae}^{†}\sigma_{ae}\right)+D\left(\rho \left(t\right)\right)\\ \; & =-i\left(H_{\gamma_{a}}\rho \left(t\right)-\rho \left(t\right)H_{\gamma_{a}}^{†}\right)+D_{13}\left(\rho \left(t\right)\right)+\gamma_{a}\sigma_{ae}\rho \left(t\right)\sigma_{ae}^{†}, \end{array}$$

where the latter term can be written as $\gamma_{a}\sigma_{ae}\rho \left(t\right)\sigma_{ae}^{†}=\rho_{ee}\left|a\rangle \langle a\right|$, i.e., corresponding to the equation ${\dot{\rho }}_{aa}=\gamma_{a}\rho_{ee}$.

The Lindblad equation can, thus, be decomposed into a set of equations involving only the lossy Λ system:

(A99) $$\begin{array}{cc} \; & {\dot{\rho }}_{11}=-iu_{p}\left(\rho_{e1}-\rho_{1e}\right)+\gamma_{1}\rho_{ee}, \end{array}$$

(A100) $$\begin{array}{cc} \; & {\dot{\rho }}_{13}=-iu_{p}\rho_{e3}+iS\rho_{1e}, \end{array}$$

(A101) $$\begin{array}{cc} \; & {\dot{\rho }}_{1e}=-iu_{p}\left(\rho_{ee}-\rho_{11}\right)+iu_{s}\rho_{13}-\frac{\gamma }{2}\rho_{1e}, \end{array}$$

(A102) $$\begin{array}{cc} \; & {\dot{\rho }}_{33}=-iu_{s}\left(\rho_{e3}-\rho_{3e}\right)+\gamma_{3}\rho_{ee}, \end{array}$$

(A103) $$\begin{array}{cc} \; & {\dot{\rho }}_{3e}=-iu_{s}\left(\rho_{ee}-\rho_{33}\right)+iu_{p}\rho_{31}-\frac{\gamma }{2}\rho_{3e}, \end{array}$$

(A104) $$\begin{array}{cc} \; & {\dot{\rho }}_{ee}=-iu_{p}\left(\rho_{1e}-\rho_{e1}\right)-iu_{s}\left(\rho_{3e}-\rho_{e3}\right)-\gamma \rho_{ee}, \end{array}$$

with the total decay rate $\gamma =\sum_{j=1,3,a}\gamma_{j}$, and a set of equations involving states |e〉 and |a〉:

(A105) $$\begin{array}{cc} \; & {\dot{\rho }}_{1a}=-iu_{p}\rho_{ea}, \end{array}$$

(A106) $$\begin{array}{cc} \; & {\dot{\rho }}_{3a}=-iu_{s}\rho_{ea}, \end{array}$$

(A107) $$\begin{array}{cc} \; & {\dot{\rho }}_{ea}=-i\left(u_{p}\rho_{1a}+u_{s}\rho_{3a}\right)-\frac{\gamma }{2}\rho_{ea}, \end{array}$$

(A108) $$\begin{array}{cc} \; & {\dot{\rho }}_{aa}=\gamma_{a}\rho_{ee}. \end{array}$$

Since we consider neither initial population in states |e〉 and |a〉 nor initial coherence involving these states (at time $t_{i}$), the two sets of equations are independent and the solution of (A105)–(A108) is $\rho_{1a}=\rho_{2a}=\rho_{ea}=0$ and:

(A109) $$\begin{array}{c} \rho_{aa}\left(t\right)=\gamma_{a}\int_{t_{i}}^{t}\rho_{ee}\left(s\right)ds \end{array}$$

determined from the knowledge of the population $\rho_{ee}\left(t\right)$ from solving the system of Equations (A99)–(A104).

The system (A99)–(A104) of interest can, thus, be reformulated, without any approximation, as the reduced Lindblad equation:

(A110) $$\begin{array}{cc} \frac{d\rho }{dt} & =-i\left(H_{\Gamma }\rho \left(t\right)-\rho \left(t\right)H_{\Gamma }^{†}\right)+D_{\Lambda }\left(\rho \left(t\right)\right), \end{array}$$

where all the operators are restricted to the Λ-system with a lossy state |e〉 of rate $\Gamma =\gamma_{a}$ associated with the effective Hamiltonian Equation (1):

(A111) $$H_{\Gamma }=\left[\begin{array}{ccc} 0 & 0 & u_{p}\\ 0 & 0 & u_{s}\\ u_{p} & u_{s} & -i\frac{\Gamma }{2} \end{array}\right],$$

and the dissipator:

(A112) $$D_{\Lambda }\left(\rho \left(t\right)\right)=\left[\begin{array}{ccc} \gamma_{1}\rho_{ee} & 0 & -\frac{\gamma_{1}+\gamma_{3}}{2}\rho_{1e}\\ 0 & \gamma_{3}\rho_{ee} & -\frac{\gamma_{1}+\gamma_{3}}{2}\rho_{3e}\\ -\frac{\gamma_{1}+\gamma_{3}}{2}\rho_{e1} & -\frac{\gamma_{1}+\gamma_{3}}{2}\rho_{e3} & -\left(\gamma_{1}+\gamma_{3}\right)\rho_{ee} \end{array}\right]$$

taking into account the decay channels within the Λ-system. This shows the relevance of the use of the lossy Hamiltonian Equation (1) for the decay operating outside the Λ-system. The system (A99)–(A104) can also be expanded in matrix form as follows:

(A113) $$\begin{array}{c} \frac{d}{dt}\left[\begin{array}{c} \rho_{11}\\ \rho_{1e}\\ \rho_{e1}\\ \rho_{ee}\\ \rho_{3e}\\ \rho_{e3}\\ \rho_{33}\\ \rho_{13}\\ \rho_{31} \end{array}\right]=\left[\begin{array}{ccccccccc} 0 & iu_{p} & -iu_{p} & \gamma_{1} & 0 & 0 & 0 & 0 & 0\\ iu_{p} & -\frac{\gamma }{2} & 0 & -iu_{p} & 0 & 0 & 0 & iu_{s} & 0\\ -iu_{p} & 0 & -\frac{\gamma }{2} & iu_{p} & 0 & 0 & 0 & 0 & -iu_{s}\\ 0 & -iu_{p} & iu_{p} & -\gamma & -iu_{s} & iu_{s} & 0 & 0 & 0\\ 0 & 0 & 0 & -iu_{s} & -\frac{\gamma }{2} & 0 & iu_{s} & 0 & iu_{p}\\ 0 & 0 & 0 & iu_{s} & 0 & -\frac{\gamma }{2} & -iu_{s} & -iu_{p} & 0\\ 0 & 0 & 0 & \gamma_{3} & iu_{s} & -iu_{s} & 0 & 0 & 0\\ 0 & iu_{s} & 0 & 0 & 0 & -iu_{p} & 0 & 0 & 0\\ 0 & 0 & -iu_{s} & 0 & iu_{p} & 0 & 0 & 0 & 0 \end{array}\right]\left[\begin{array}{c} \rho_{11}\\ \rho_{1e}\\ \rho_{e1}\\ \rho_{ee}\\ \rho_{3e}\\ \rho_{e3}\\ \rho_{33}\\ \rho_{13}\\ \rho_{31} \end{array}\right]. \end{array}$$
